# Cysteine and iron accelerate the formation of ribose-5-phosphate, providing insights into the evolutionary origins of the metabolic network structure

**DOI:** 10.1371/journal.pbio.3001468

**Published:** 2021-12-03

**Authors:** Gabriel Piedrafita, Sreejith J. Varma, Cecilia Castro, Christoph B. Messner, Lukasz Szyrwiel, Julian L. Griffin, Markus Ralser

**Affiliations:** 1 Department of Biochemistry and Cambridge Systems Biology Centre, University of Cambridge, United Kingdom; 2 Department of Biochemistry, Charité Universitätsmedizin Berlin, Berlin, Germany; 3 The Molecular Biology of Metabolism Laboratory, The Francis Crick Institute, London, United Kingdom; 4 The Rowett Institute, The University of Aberdeen, Aberdeen, United Kingdom; University College London, UNITED KINGDOM

## Abstract

The structure of the metabolic network is highly conserved, but we know little about its evolutionary origins. Key for explaining the early evolution of metabolism is solving a chicken–egg dilemma, which describes that enzymes are made from the very same molecules they produce. The recent discovery of several nonenzymatic reaction sequences that topologically resemble central metabolism has provided experimental support for a “metabolism first” theory, in which at least part of the extant metabolic network emerged on the basis of nonenzymatic reactions. But how could evolution kick-start on the basis of a metal catalyzed reaction sequence, and how could the structure of nonenzymatic reaction sequences be imprinted on the metabolic network to remain conserved for billions of years? We performed an in vitro screening where we add the simplest components of metabolic enzymes, proteinogenic amino acids, to a nonenzymatic, iron-driven reaction network that resembles glycolysis and the pentose phosphate pathway (PPP). We observe that the presence of the amino acids enhanced several of the nonenzymatic reactions. Particular attention was triggered by a reaction that resembles a rate-limiting step in the oxidative PPP. A prebiotically available, proteinogenic amino acid cysteine accelerated the formation of RNA nucleoside precursor ribose-5-phosphate from 6-phosphogluconate. We report that iron and cysteine interact and have additive effects on the reaction rate so that ribose-5-phosphate forms at high specificity under mild, metabolism typical temperature and environmental conditions. We speculate that accelerating effects of amino acids on rate-limiting nonenzymatic reactions could have facilitated a stepwise enzymatization of nonenzymatic reaction sequences, imprinting their structure on the evolving metabolic network.

## Abstract 2

The evolutionary origins of metabolism are largely unknown. This study shows that the prebiotically available proteinogenic amino acid cysteine can promote the metabolism-like rate-limiting formation of ribose-5-phosphate, suggesting that early metabolic pathways could have emerged thought the stepwise enzymatization of non-enzymatic reaction sequences.

## Introduction

Cellular metabolism is constituted by a highly conserved and ancient biochemical system, the metabolic network. The size and complexity of the metabolic network are major constraints in the evolution of cells. In prokaryotic cells, this network typically involves up to two-thirds of the genes encoded in their genome and can consist of several hundred enzymes that interconvert thousands of different metabolites [1–5]. Despite its huge size and conservation, the topological organization of the network builds on individual reaction components that are, on their own, relatively simple. Catalysis is conducted by only 6 classes of enzymes that make use of the same cofactors for catalysis, energy, and electron transfer, while covering a small fraction of the possible chemical space [6,7].

The evolutionary origins of the metabolic network remain largely unknown [8–14]. One of the earliest explanations for the early evolution of metabolic pathways was given by a model in which simple ribozymes could have primed the evolution of metabolism [15–17]. However, while the role of RNA in the evolution of transcription and translation is broadly accepted, the genomic era has so far not been able to associate ribozyme properties to rationalize the pathway structures of central metabolism [18]. Instead, metabolism-like nonenzymatic reactions can be promoted by single amino acids, nucleotides, and metal ions. Not only are these prebiotically available, but they also catalyze the majority of metabolic reactions in modern cells. Occam’s razor hence suggests that the metabolic network structure was directly selected alongside the evolution of protein-based enzymes that sequestered metal ions, nucleotides, and other small organic molecules as cofactors [13,19–22].

A second difficulty in the origins of metabolism is to explain the event of the multistep reaction sequences that are characteristic for the metabolic network. These could emerge from autocatalytic chemical cycles, but these are not evolvable [23]. Second, as early as in 1945, Horowitz described another difficulty in explaining the advent of enzymatic pathway topologies by Darwinian evolution as a chicken–egg dilemma, also previously referred to as the “end product problem” [12,24]. The problem emerges, as Darwinian pressure can only select for a functional product, the “end product” of an enzymatic pathway, however, not for intermediates that do not provide any advantage on their own. These intermediates need, however, to form at sufficient flux, before the advantage-providing “end product” forming enzyme can be selected. One model overcomes this problem by suggesting that all intermediates could have had functional relevance early in the evolution [25]. This situation is, however, not reflected in the modern metabolic network. Other models such as the patchwork hypothesis and the semienzymatic models invoke the appearance of a few nonspecific enzymes early on during evolution that underwent gene duplication to be able to transform a broad range of substrates [26,27] or duplicated enzymes transforming metabolites leaking from existing pathways or nonenzymatic reactions [28].

**Fig 1:**
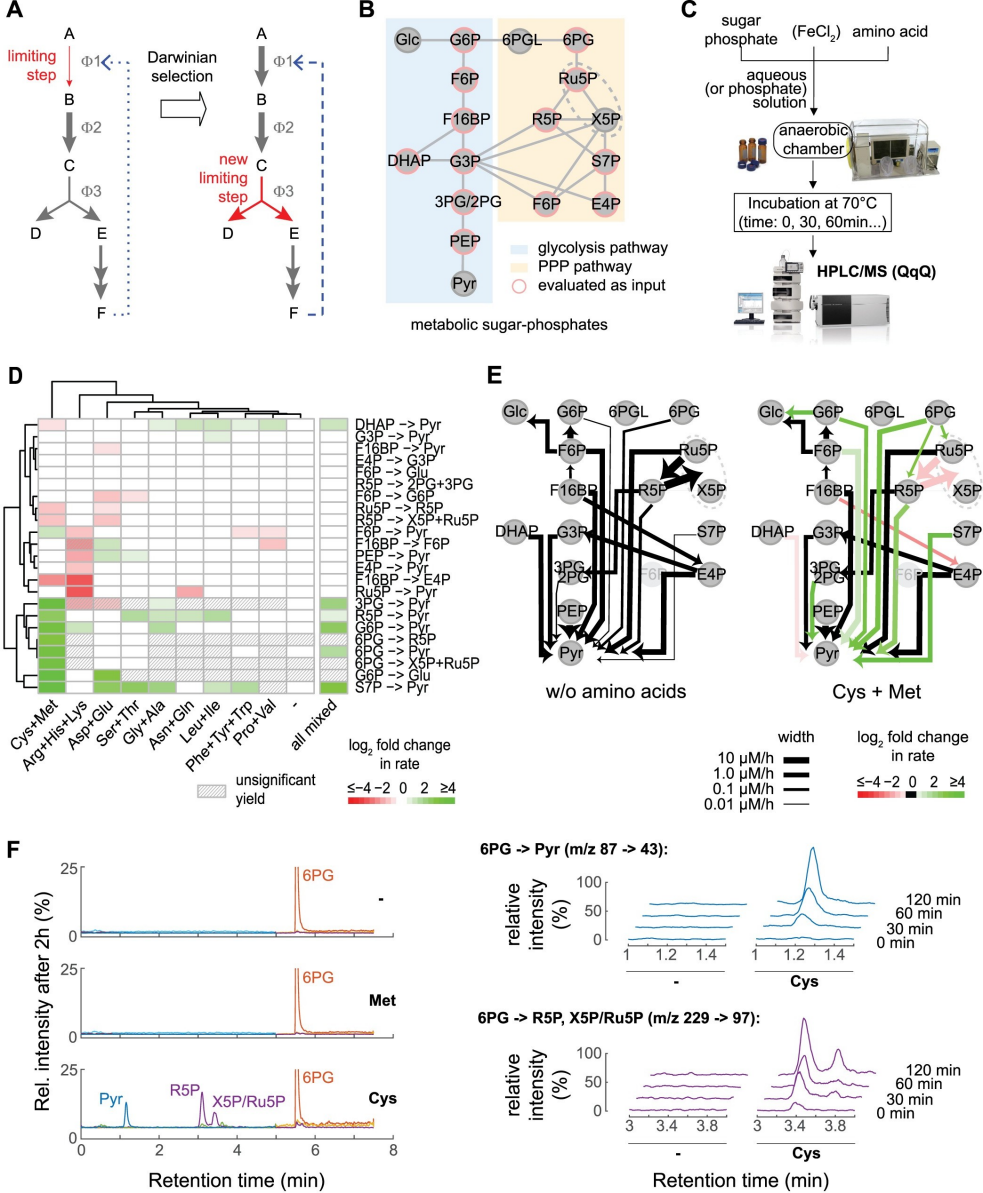
Multiple amino acids, in particular cysteine, accelerate or slow nonenzymatic interconversions between glycolytic and PPP sugar phosphates. **(A)** Hypothesis of early metabolic pathway evolution through stepwise enzymatization of a nonenzymatic chemical network. Selective pressure to increase the formation of a metabolite acts on the slowest or least efficient (rate-limiting) step of the chemical network that forms the product (ɸ1, red). A primitive enzyme that acts on this rate-limiting step (dashed blue arrow) increases the performance of the entire pathway. Improvements on this step would eventually lead to another reaction (ɸ3) becoming the new rate-limiting step, and this process keeps repeating to give rise to an enzymatic pathway. The stepwise enzymatization, as proposed by this model, would imprint the structure of the nonenzymatic reaction sequence on the evolving metabolic network. **(B, C)** Experimental setting to test if amino acids, as the simplest building blocks of enzymes, could provide the chemical properties necessary to make the model as in (A) viable. (B) Network topology of metabolic sugar phosphate interconversions from glycolysis and PPP, whose metabolites interconvert nonenzymatically in the presence of Fe(II) [34,44]. Enzymes and cosubstrates were omitted in this graph, and only sugar phosphates analyzed in this study are displayed (those 12 used as input appear surrounded in red). (C) Experimental procedure to test the influence of proteinogenic amino acids on metabolism-like nonenzymatic interconversions. Each sugar phosphate was dissolved in aqueous (or 50 mM phosphate) solution in the presence or absence of single amino acids. Samples were incubated at 70°C in glass vials sealed in an anaerobic chamber (picture courtesy of Coy Laboratory Products) before targeted detection and quantification of sugar phosphate products (from all those in (B)) using LC–SRM (picture courtesy of Agilent Technologies, Inc.). **(D)** Amino acid effects on individual production rates (rows), illustrated as a heat map, expressing log fold changes versus a control case without amino acids (columns). Striped cells denote conditions where the product was not detectable at a significant concentration, and only significant rate changes are colored (Mann–Whitney *U* test, α = 0.1). Individual sugar phosphates were incubated at a concentration of 100 μM, at 70°C, in aqueous solutions containing single, proteinogenic amino acids, grouped, according to functional similarity, at a total concentration of 400 μM. “All mixed” corresponds to a condition where all 20 amino acids were present at a total concentration of 400 μM. Data shown correspond with the average from *N* = 3 independent experiments per condition (see S1 Data). **(E)** The effect of cysteine/methionine on nonenzymatic metabolism–like interconversions of central sugar phosphates. The summary diagram illustrates nonenzymatic transformations detected in the aqueous solution (left panel) compared with those in the presence of cysteine and methionine (right panel). The width of the arrows illustrates the detected average reaction rates from *N* = 3 independent experiments (see S1 and S2 Data and Materials and methods). Arrow width for nonaffected E4P -> G3P reaction is downscaled to half-size for visual purposes. The same color coding as in (A) is applied to transformations with significantly different rates (either higher = green or lower = red) in the cysteine + methionine case versus control (black = nonsig. change). **(F)** Cysteine promotes metabolism-like reactivity of 6-phosphogluconate (6PG), with R5P being the dominant reaction product. Representative chromatograms obtained by LC–SRM after 2 hours incubation of 100 μM 6PG in control conditions without amino acids (−), in the presence of 200 μM methionine, or with 200 μM cysteine, revealing that cysteine is responsible for facilitating the formation of different products (*N* = 3). Right panels: Detailed time courses in the formation of pyruvate and pentose phosphate sugars (SRM transitions displayed) (see S1 Data). Note that R5P is referred for simplicity, even if R5P would be indistinguishable from its diastereomer arabinose 5-phosphate. 3PG/2PG, 3-phosphoglycerate and 2-phosphoglycerate; 6PG, 6-phosphogluconate; 6PGL, 6-phosphogluconolactone; DHAP, dihydroxyacetone phosphate; E4P, erythrose 4-phosphate; F6P, fructose 6-phosphate; F16BP, fructose 1,6-bisphosphate; Glc, glucose; G3P, glyceraldehyde 3-phosphate; G6P, glucose 6-phosphate; LC–SRM, liquid chromatography–selective reaction monitoring; PEP, phosphoenolpyruvate; PPP, pentose phosphate pathway; Pyr, pyruvate; R5P, ribose-5-phosphate; Ru5P, ribulose 5-phosphate; S7P, sedoheptulose 7-phosphate; X5P, xylulose 5-phosphate.

More recently, the discovery of metabolism-like nonenzymatic chemical reactions that are driven by simple, prebiotically abundant metal ions has shed a new light on the evolution of early enzymes, as well as suggests an alternative solution to the end product problem. In the absence of enzymes, intermediates could simply be formed by nonenzymatic reactions [12]. Nonenzymatic reactions also relax the problem of the nonevolvability of autocatalytic chemical cycles [23], if the chemical cycles are not evolving on their own, but rather act as a “template” that kick-starts the evolution of metabolism. Such a scenario indeed is favored by metabolic control theory, which describes that in a metabolic network, some reactions are the most limiting for flux and are hence exposed to the highest selection pressure during evolution. If the rate-limiting reaction is improved, the “advantage-providing” metabolite is produced at a higher rate or efficiency, even if it is downstream. Reflecting on the origins of metabolism, this means that the acceleration of one reaction at a time can improve the flux to the end product also in a multistep reaction sequence [29–31]. Taken together, the model suggests a “stepwise enzymatization.” The first, simple enzymes would be selected for accelerating the most rate-limiting reactions of a nonenzymatic network. Once this reaction is accelerated to the extent it is no longer rate limiting, the selection pressure is shifted to the next limiting reaction. Step by step, a nonenzymatic reaction sequence would be populated by enzymes (Fig 1A).

The stepwise enzymatization model generates a series of testable hypotheses. First, it requires the existence of nonenzymatic, or environmental, reaction sequences that resemble modern metabolic pathway structures, a continuity scenario already put forward by de Duve and Morowitz [32,33]. Several such networks have been described recently. The first such network resembled glycolysis and the pentose phosphate pathway (PPP) and topologically related pathways such as the Entner–Doudoroff pathway, which share several of their metabolites [34]. This discovery was followed by the description of nonenzymatic reaction sequences that also resemble parts of gluconeogenesis, the oxidative and the reductive Krebs cycle, the Wood–Ljungdahl pathway and the S-Adenosylmethionine pathway. In aggregate, the network of nonenzymatic and iron-driven reactions capture many key features of the reaction topology of the extant central metabolic network [22,35–42].

Second, the stepwise enzymatization model requires all the reactions to occur in the same reaction condition, i.e., without condition step changes, so that the selection process can occur. Indeed, most of the nonenzymatic glycolytic and PPP-like reactions are accelerated and gain efficiency upon the addition of ferrous iron, the most abundant transition metal in Archean sediment [34,43,44].

In this study, we test another condition for the stepwise enzymatization model. In order for a metal catalyzed reaction network to form a basis for the evolution of metabolism, simple components that later form enzymes, amino acids, or peptides need to interact with the reaction sequence and favorably change its reaction properties. We chose the nonenzymatic network of glycolytic and PPP-like reactions [34,44]. We then systematically combined the metabolites with the 20 universal proteinogenic amino acids. Then, we followed the approximately 200 possible carbohydrate interconversion reactions by quantitative selective reaction monitoring, a highly sensitive mass spectrometry technique, which can detect the formation of sugar phosphates also in the presence of ferrous iron, the dominant Archean sediment transition metal [45,46] (Fig 1B and 1C). We discover that amino acids have a broad impact on the glycolysis and PPP-like nonenzymatic metabolism–like reactions occurring in water. One reaction caught our attention in particular. The formation of ribose-5-phosphate from 6-phosphogluconate is rate limiting in the extant PPP, but no nonenzymatic ration that resembles this interconversion is detected in water. However, the reaction proceeds both in the presence of iron and amino acids. Cysteine, a prebiotically available amino acid, has additive effects on the reaction rate in the iron rich milieu and interacts with iron, forming a ternary complex of the substrate, iron, and the amino acid. We show that in the presence of iron and cysteine, metabolism-like ribose-5-phosphate formation occurs even at 20°C and at high specificity.

## Results

### Proteinogenic amino acids impact nonenzymatic metabolism–like interconversions between metabolites of glycolysis and the PPP

An in vitro screening was devised in order to assess the influence of proteinogenic amino acids on the metabolism-like nonenzymatic reactivity of glycolytic and PPP intermediates [47,48]. A total of 12 commercially available sugar phosphate intermediates—7 belonging to glycolysis (Embden–Meyerhof–Parnas (EMP) pathway; 4 of those shared with the Entner–Doudoroff pathway) and 5 to the PPP—were dissolved at a concentration of 100 μM in different aqueous solutions containing proteinogenic amino acids (Fig 1B and 1C). Amino acids were grouped in sets of 2 or 3 according to chemical similarity of the side chain and added at a collective total concentration of 400 μM.

To account for the situation that early metabolic enzymes likely produced lower amounts of metabolites compared to highly evolved, modern metabolic enzymes [49], we chose a sensitive liquid chromatography–selective reaction monitoring (LC–SRM) method that reliably detects the metabolites even at concentrations that are lower than their typical cellular levels [44]. LC–SRM overcomes a limitation of origin-of-metabolism research, caused by ^1^H-NMR spectroscopy, which is frequently applied in this field due to its high identification precision. ^1^H-NMR, however, suffers from signal suppression when Archean sediment typical iron concentrations are present in the reaction mixtures and hence fails to detect a broad spectrum of transient, iron-promoted, metabolism-like nonenzymatic reactions that are increasingly considered as a basis for early metabolic evolution [45,46]. Samples were prepared in an artificial nitrogen atmosphere to simulate low oxygen concentrations that keep iron in its reduced, water-soluble form [43]. The mixtures were heated to 70°C, a compromise previously set to achieve detectable nonenzymatic reaction rates in water while still being within the temperature range where many hyperthermophiles relying on the EMP pathway or structurally similar pathways are known to grow [50]. To capture the dynamic behavior of the nonenzymatic network in which most metabolites are formed transiently (i.e., as in metabolism, most metabolites do not accumulate to high concentrations but constantly react further), we sampled at multiple time points over a total experimental time of 6 hours. Once sugar phosphate products were detected and quantified, we reconstructed the kinetics of metabolite formation and consumption.

Several sugar phosphate products formed and indicated a metabolism-like topology of a nonenzymatic reaction network (Fig 1D and 1E; see S1 and S2 Data and Materials and methods). Overall, a total of 23 nonenzymatic transformations that resemble reactivity as occurring within glycolysis and the PPP were detected (a similar number as we reported previously in a slightly different setup [34]). The presence of multiple amino acids did have a noticeable impact on the reaction rates (Fig 1D, S1 Fig). We observed both the acceleration and the deceleration of several nonenzymatic conversions, with typical rate fold changes in the range 0.25 to 4.0. Overall, 13 metabolite interconversions were significantly enhanced by at least 1 group of amino acids, when compared to a control case without amino acids (Mann–Whitney *U* test, α = 0.10).

As one can expect from the nature of chemical reactions, a rate can be changed either by the direct interaction, i.e., of an amino acid with the sugar phosphates, or by indirect effects, like changes in pH. A number of reactions were indeed accelerated or slowed by the positively charged amino acids (arginine, histidine, and lysine) as well as by negatively charged ones (aspartate and glutamate). These rate changes were hence possibly explained by their ability to change their protonation level in response to shifts in the pH, an important factor in the nonenzymatic reactivity of sugar phosphates [44] (S1 Fig). However, the most prominent changes occur with the sulfur-containing amino acids, cysteine, and methionine (Fig 1D and 1E, S1 Fig). A total of 8 out of 23 nonenzymatic glycolysis and PPP-like reactions were 6-fold accelerated over their rate in water with this group of amino acids.

### Ribose-5-phosphate forms from 6-phosphogluconate in the presence of cysteine as in the extant PPP

Sulfur-containing amino acids had their most striking effect on the nonenzymatic reactivity of 6-phosphogluconic acid (6PG), a central metabolite of the PPP. In many modern cells, 6PG participates in a rate-limiting metabolic reaction that forms ribose-5-phosphate, which is then further metabolized and forms, among others, the RNA and, subsequently, the DNA backbone. In water, and in the presence of all other amino acids, 6-phosphogluconate was stable and did not yield any significant concentration of ribose-5-phosphate or other intermediates of the PPP (Fig 1D). In the presence of the sulfur-containing amino acids, however, 6-phosphogluconate was converted. The metabolites detected at highest concentration were pyruvate and the pentose phosphate sugars ribose-5-phosphate and ribulose-5-phosphate/xylulose-5-phosphate (please note that isomers are indistinguishable from each other by the LC–SRM method if they have the same chromatographic retention time) (Fig 1E).

**Fig 2:**
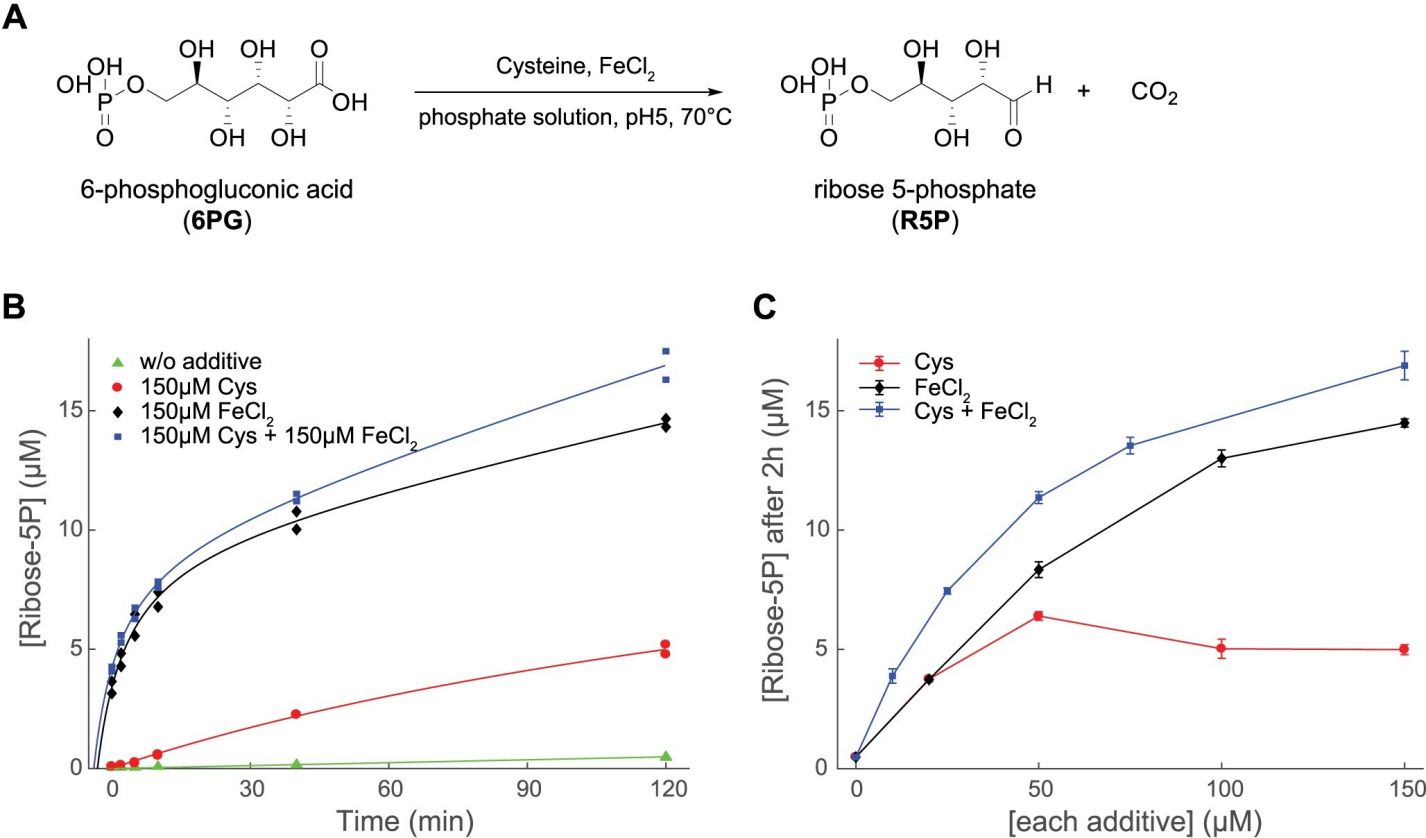
Nonenzymatic formation of R5P from 6-phosphogluconate. **(A)** The nonenzymatic reaction of conversion of 6-phosphogluconate to R5P. **(B, C)** The formation of R5P from 800 μM 6PG in 50 mM phosphate solution at pH 5 was monitored at 70°C for (B) different time points (*N* = 2) (see S1 Data) and (C) different concentrations of cysteine, Fe(II), or both species (combined in a molar ratio 1:1). Data shown as mean ± SD (*N* ≥ 2 per condition) (see S1 Data). 6PG, 6-phosphogluconic acid; R5P, ribose-5-phosphate.

We next determined whether cysteine, methionine, or both sulfur-containing species were responsible for enhancing 6-phosphogluconate transformations. We found that the effects were exclusively explained by cysteine (Fig 1F). As reaction rates in nonenzymatic PPP reactions are strictly pH dependent [44], we tested product formation under different pH regimes in order to set optimal conditions for further characterization of ribose-5-phosphate formation (and/or that of some of its isomers, like arabinose 5-phosphate, which would be indistinguishable from ribose-5-phosphate). A total of 50 mM phosphate solutions spanning pH values between 3 and 9 were used. We obtained strongly pH-dependent reaction rate profiles that were distinct for the different products detected, making prominent changes evident and relatively narrow optimal pH values under cysteine conditions, suggestive of catalysis (S2 Fig). Other intermediates of the PPP that were not detected before under unbuffered water conditions (erythrose 4-phosphate and 6-phosphogluconolactone) were now detected under certain pH conditions (Supporting information Note in S1 Text). Maximum quantities of ribose-5-phosphate and its isomers were observed under mild acidic conditions. Therefore, further experimental conditions were set at pH 5, where cysteine is also stabilized (Fig 2A, S3 Fig).

In order to get insights into the nature of this reaction, we first tested a panel of sulfur-containing cysteine analogues to explore the impact of chirality, chain length, functional group, and oxidation state on the enhancement of the nonenzymatic reaction from 6-phosphogluconate. Considering closest similarity and commercial availability, we chose the following molecules: L- and D-forms of cysteine (to test for chirality effects), D/L-homocysteine and reduced glutathione (for molecular or chain-size effects), 3-mercaptopropionic acid, cysteamine, β-mercaptoethanol (for differing from cysteine just in the carboxylic, the amino group or both), and L-cystine, cysteine sulfonic acid, and oxidized glutathione (oxidized forms of cysteine thiol group). Ribose-5-phosphate formation rate was most affected by the oxidation state of the sulfur and, to a lesser extent, by the nature of the other pendant functional groups (S4 Fig). Oxidized sulfur groups did not produce any significant amount of ribose-5-phosphate, indicating the thiol group (R-SH) was crucial for the activity. In fact, serine, unlike cysteine, did not display any acceleration on product formation when compared to control conditions without amino acids (S4A Fig). The carboxyl and amino groups of cysteine appeared to play relevant roles too as important modulatory factors, suggesting a relatively narrow chemical spectrum for drivers of these interconversions. We reasoned that the fact of an optimum reaction rate at pH 5 may in part respond to cysteine stability reasons (it is prone to oxidation—making the reaction less efficient—at higher, alkaline pH conditions; S3 Fig) but could be related too to its closeness to cysteine isoelectric point, where the zwitter ionic state of the amino acid could be involved in tethering different components together. It is believed that in an hypothetical scenario of evolution toward the emergence of peptide bonds, these carboxylic and amine group effects from cysteine could have been replaced by terminal or side-chain residues from other amino acids like glutamic acid or lysine, as the tripeptide reduced glutathione retained similar activity as cysteine (S4 Fig).

Finally, in order to assess the possible role that the availability of molecular oxygen could play in modulating the reaction rate, we tested the product formation in the ambient, aerobic atmosphere. The yield of ribose-5-phosphate formed from 6-phosphogluconate in the presence of cysteine in the aerobic environment was similar to that when incubated in the anaerobic chamber (i.e., 8 to 12 ppm O_2_), arguing that the formation of ribose-5-phosphate was robust despite approximately 1,000-fold difference in O_2_ concentration between these conditions at the given timescale, when cysteine remains relatively stable (S1 Table). The effect of oxygen is considered to be detrimental for the reaction rate at longer timescales or when cysteine is combined with the metal Fe(II) that quickly oxidizes to Fe(III) (see below; S1 Table).

### Cysteine and iron form a reactive species that complement each other

Iron is the dominating transition metal in Archean sediment; it is water soluble in its reduced form, and the sediment record shows it was highly concentrated in the Archean waters [43]. In today’s oxygenated atmosphere, iron concentrations are much lower, but even today, it requires chelating agents to deprive it of environmental iron. The consequence is that iron is ubiquitously present in living systems. Indeed, iron is an essential component of metabolism and dominates the spectrum of metabolism-like nonenzymatic reactions discovered to date, including the aforementioned transformation of glucose-6-phosphate to ribose-5-phosphate [34,44]. A reasonable assumption is hence that metabolism evolved in the presence of iron and that the evolution of metabolic enzymes had to accelerate the reactions above their rate or specificity achieved with environmental iron concentrations. We hence tested whether cysteine could enhance ribose-5-phosphate also in an environment in which the transformation of glucose-6-phosphate to ribose-5-phosphate already occurs on the basis of an environment typical iron concentration. We first compared the formation of ribose-5-phosphate from 6-phosphogluconate in the presence of Fe(II) and cysteine supplied individually. Moreover, 800 μM 6-phosphogluconate was incubated in 50 mM phosphate solutions containing either FeCl_2_ or cysteine. Ribose-5-phosphate formed both in the presence of Fe(II) and cysteine, respectively (Fig 2), and both reactions showed a similar pH-dependent reaction profile, only differing in slightly distinct optimal pH values (S2 Fig). The maximum formation rate for ribose-5-phosphate occurred at pH 5 and was higher with Fe(II) than with cysteine (Fig 2B). Furthermore, the activity of cysteine slowed at concentrations above 100 μM, an effect not observed for iron (Fig 2C). We then tested cysteine and iron at diverse molar ratios and total concentration values. The maximum of ribose-5-phosphate production was obtained when iron and cysteine were combined, indicating that the presence of cysteine does further increase ribose-5-phosphate formation also in iron-rich conditions, and vice versa (Fig 2B and 2C). Indeed, the data from different molar ratios of these 2 species suggest that the combined net contribution is consistent with a simple model of weighted additive effects (S5 Fig). Hence, the co-occurrence of cysteine in an evolutionary scenario characterized by the presence of environmental iron would accelerate further the formation of ribose-5-phosphate.

**Fig 3:**
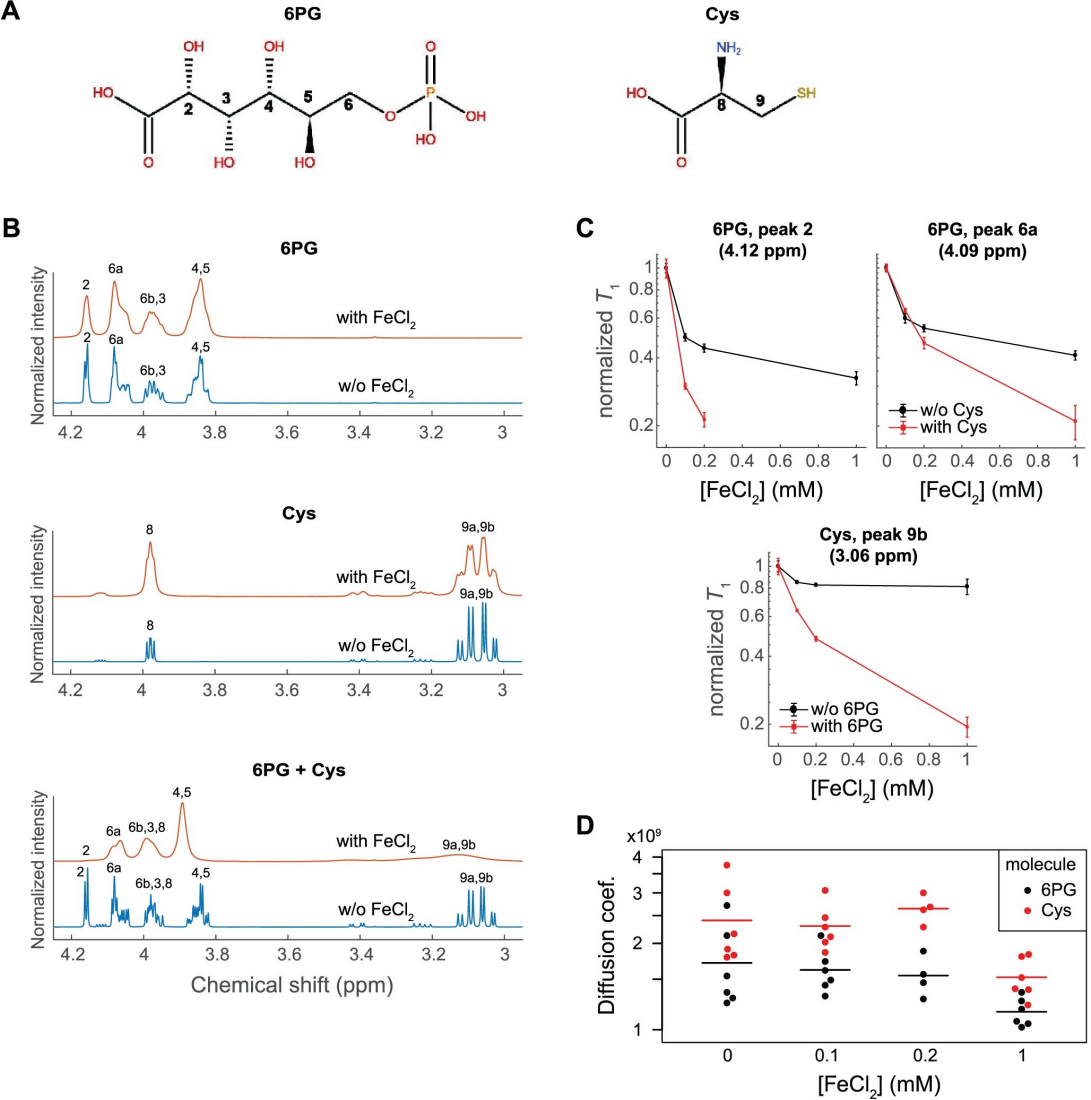
Complementary interactions of cysteine and Fe(II) with 6PG. **(A)** 6-phosphogluconate and cysteine molecular structures, indicating positions of resonant protons. **(B)** Representative ^1^H-NMR spectra of 6PG alone, cysteine alone, or the combination of both (20 mM of each analyte) in 50 mM phosphate pH 5/D_2_O solutions, either in the absence or presence of 1 mM FeCl_2_. Peak numbers correspond with proton labels in (A). The massive disruption and suppression of the ^1^H signal peaks upon addition of Fe(II) to the binary mixture suggests a coordination between cysteine and 6PG in Fe(II) binding. Spectra are shown normalized to the maximum peak intensity per spectrum (*N* ≥ 3) (see S1 Data). **(C)** T_1_ relaxation time scaling of 6PG resonant protons near carboxylic (peak 2; 4.12 ppm) and phosphate (peak 6a; 4.09 ppm) groups and cysteine proton (peak 9b; 3.06 ppm) signals with increasing Fe(II) concentrations when analyzed separately versus in combination (conditions as in (B)). Data shown as mean ± SD from *N* = 3 independent experiments (see S1 Data). **(D)** Diffusion coefficients measured by DOSY for cysteine and 6PG molecules (20 mM each) when co-incubated with a mixture of increasing concentrations of Fe(II) and constant, saturating concentrations of phenanthroline, a strong, nonchelating Fe-binding complex. Both molecules showed a noisy but significant decrease in diffusion, a further indication of Fe(II) binding. Individual measurements (dots) and mean (horizontal line) from *N* ≥ 4 experiments (see S1 Data). 6PG, 6-phosphogluconic acid; DOSY, diffusion ordered spectroscopy; R5P, ribose-5-phosphate.

Since cysteine and iron have a strong binding affinity and are well known to form a variety of complexes that regulate protein function, we investigated if there was any structural indication of a specific combined interaction with 6-phosphogluconate. For this, we made use of ^1^H-NMR spectroscopy, taking advantage of the property that Fe(II) is paramagnetic and it distorts and suppresses the signal intensities of closely proximal protons from adjacent molecules, in part by reducing the spin–lattice relaxation times of coupled nuclei (*T*_1_) (Fig 3; Materials and methods). Treating 20 mM 6-phosphogluconate in 50 mM phosphate D_2_O solution at pH 5 with 1mM FeCl_2_ revealed a general smoothing and broadening of the different ^1^H-NMR spectral peaks of 6PG, indicative of an interaction of the sugar phosphate with the metal ion (Fig 3B). Similarly, cysteine demonstrated a fairly strong interaction with Fe(II) at pH 5 (^1^H-NMR spectral peaks at 3.97 ppm and 3.06 ppm) even at relatively low molar fractions of Fe (II) (e.g., with 1 mM Fe(II)) (Fig 3B). In either case, it was not possible to discriminate the interaction site with the metal at the level of a specific functional group given the close spatial proximity between the resonant protons. Strikingly, under the same concentrations as above, Fe(II) dramatically suppressed resonances from both cysteine and 6-phosphogluconate spectra when these species were mixed together (Fig 3B). This indicated the much tighter interaction achieved with the 3-component system, viz 6-phosphogluconate, iron, and cysteine. In particular, the peak from proton bound to C2 of 6PG (chemical shift at 4.12 ppm) was the most affected one (Fig 3B), consistent with interactions occurring predominantly proximal to the carboxylic group, the group which is to be converted into aldehyde in the transformation of 6PG into R5P (Fig 2A). These observations were further supported by the *T*_1_ relaxation time analyses: Shortening of cysteine *T*_1_ relaxation time by Fe(II) became consistently more pronounced in the presence of 6-phosphogluconate, and vice versa, even if differences of proton behavior along a given molecule were subtle (Fig 3C, Tables B–D in S1 Text). Conversely, no significant or much weaker effects were attained when cysteine was substituted with methionine or when 6-phosphogluconate was replaced by the analogue glucose 6-phosphate (S6 Fig, Tables E–H in S1 Text), supporting the validity of the ^1^H-NMR approach showing the specificity of the interaction.

Altogether, these data would evoke a cooperative interaction between cysteine, Fe(II) and the 6-phosphogluconate, which would form an assembly that could presumably underpin the catalysis. Molecular diffusion measurements carried out by diffusion ordered spectroscopy (DOSY) ^1^H-NMR spectroscopy showed a statistically significant decrease (Mann–Whitney *U* test, α = 0.05) in the diffusion coefficients of both 6-phosphogluconate and cysteine when these were exposed simultaneously to increasing concentrations of Fe(II) in the presence of a relatively bulky, nonchelating Fe(II) ligand such as phenanthroline (Fig 3D). This constituted further evidence pointing toward the formation of at least a transient reaction–favoring assembly. It should be noted that the concentration of product formed in the reaction mixture was too small to be detected by ^1^H-NMR, a technique which has a limited sensitivity (especially in the presence of iron), so that reaction monitoring was limited to the much more sensitive LC–SRM technology.

### Thermal-dependent reaction specificity of Cys:Fe-driven ribose-5-phosphate formation

At 70°C, temperature is assumed to be a major driver of the net reaction kinetics. There are plausible arguments that suggest that the origin of metabolism could have happened under thermophilic conditions, as thermodynamic barriers are easier to overcome [51,52]. However, the evolution of the extant metabolic network must have also involved typical physiological temperatures, as the metabolic network contains a set of essential metabolites—some indeed are major metabolic hubs like 3-phosphoglycerate (3PG)—which are highly unstable at elevated temperature. 3PG, for instance, decomposes at 70°C within seconds, yet is an essential part of the metabolic network, not only of mesophiles but also of thermophiles [34]. The 3PG example indeed shows that metabolite stability and high reaction yields are not essential requirements for metabolic evolution.

**Fig 4:**
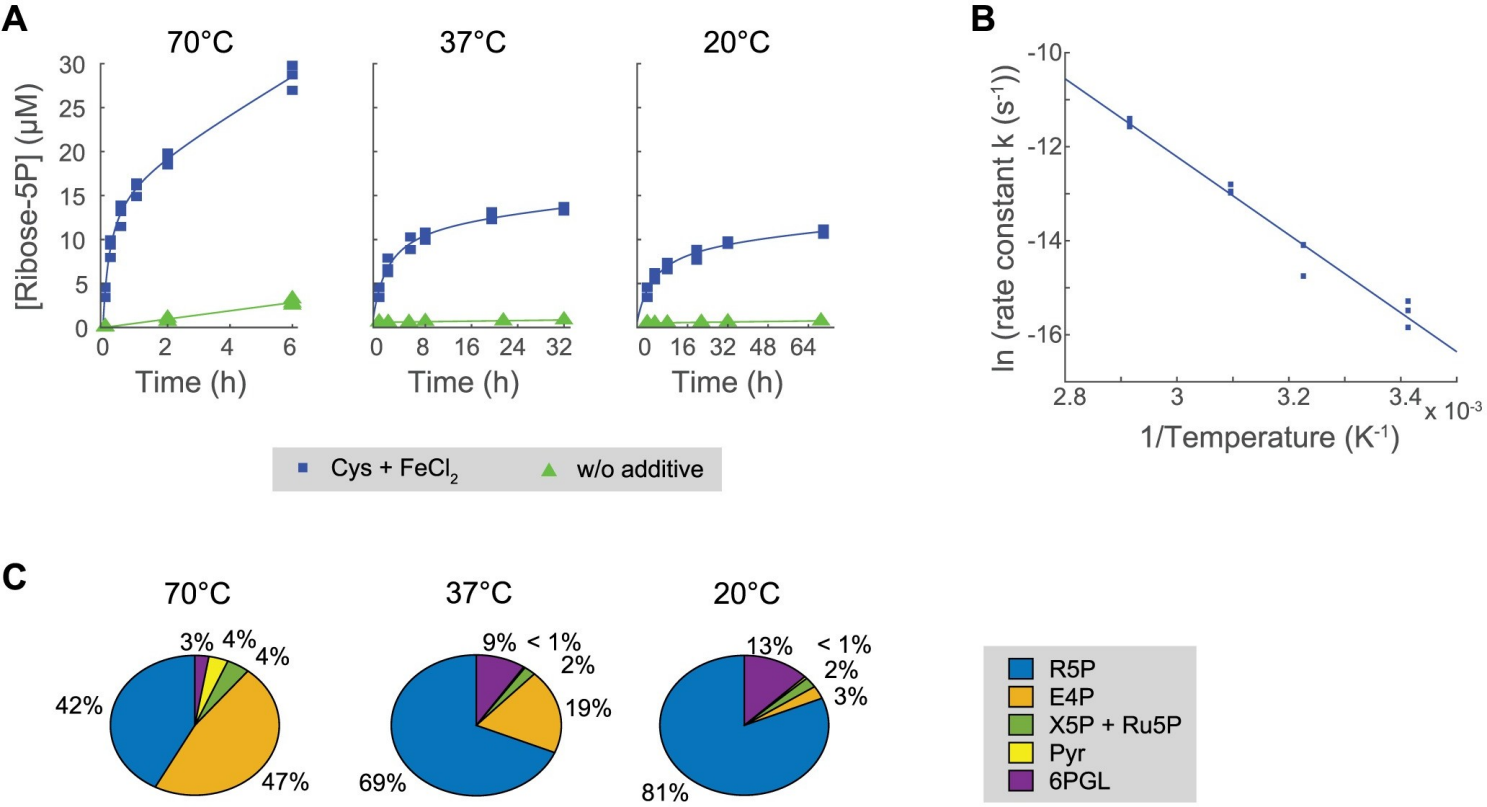
Specific metabolism-like nonenzymatic R5P formation at different temperatures. **(A)** R5P concentration over time, formed upon the incubation of 800 μM 6PG in 50 mM phosphate solution pH 5 at different temperatures in the presence of 75 μM cysteine and 75 μM FeCl_2_. Notice that time ranges were adapted according to the temperature scaling kinetics. Experimental data (points; *N* = 3) conform to hyperbolic fits (lines). Trends without the additives are shown for comparison (see S1 Data). **(B)** Initial rate constants calculated from (A) scale exponentially with the absolute temperature, following Arrhenius’ equation. The linear slope in the plot of log-transformed values gives a value for the activation energy E_a_ of 69 kJ mol^−1^ (see S1 Data). **(C)** Long-term product specificity at different temperatures. Relative concentrations of the different detected sugar phosphate products are shown for the last recorded time point per temperature condition from the experiments in (A) (average from *N* = 3 experiments) (see S1 Data). R5P, ribose-5-phosphate.

To test for the nonenzymatic formation of ribose-5-phosphate at different temperatures, we dissolved 800 μM 6-phosphogluconate in a 50 mM phosphate solution at pH 5. Upon adding 75 μM cysteine and 75 μM Fe(II), the solution was incubated for up to 3 days, depending on temperature, to allow enough time for detectable product formation. Cysteine and Fe(II) did promote the formation of ribose-5-phosphate in a whole range of mild temperatures, even though the reaction rate slowed with decreasing temperatures, in agreement with the Arrhenius equation (Fig 4A and 4B). The least squaresfit yielded a value for the activation energy *E*_a_ of 69 kJ mol^−1^ (±8; 95% confidence bounds). For the same range of temperatures, only very limited formation of ribose-5-phosphate was observed in the absence of the amino acid and the metal, due to a higher energy barrier, estimated to be around 87 kJ mol^−1^ (Fig 4A).

Interestingly, temperature-driven changes in reaction rate inversely correlated with an increased specificity of forming the signal corresponding to ribose-5-phosphate (Fig 4C, S7 Fig). While 42% of all 6-phosphogluconate–derived products corresponded to forming the pentose-5-phosphate at late time points at 70°C, this value increased to 81% at 20°C. The relative proportion of other quantified reaction products such as xylulose/ribulose 5-phosphate, pyruvate, and, especially, erythrose 5-phosphate diminished when decreasing the temperature. Only 6-phosphogluconolactone was more abundant at lower temperatures, probably due to the stability of the cyclic structure at lower temperatures, thereby trapping the reaction at this intermediate. In consequence, the relative loss of interconversion rate efficiency was compensated by an increased specificity for ribose-5-phosphate formation at low temperatures.

## Discussion

The historical biochemistry textbook has primarily explained the function of metabolism with the need for the biosynthesis of the cellular building blocks, like amino acids, nucleotides, and lipids. This view has put specific emphasis on what has become known as “anabolism,” the biosynthesis of biomolecules. If metabolism had no other function than providing these biomolecules, a variety of separate chemical processes would have hence contributed to the early forms of the metabolic network, as long as they produced the products required. Consequently, many investigations into the origins of metabolism during abiogenesis have focused on generating nucleotides, amino acids, and other biomolecules at a high concentration [20,53]. To generate high yields of the products and intermediate reactions, chemical studies into the origins of life have often considered chemical reaction sequences that show very little resemblance with metabolic pathways [54].

However, the detailed study into metabolism’s topology, given a vast amount of information that has become available about biochemical systems, draws a more differentiated picture. For the evolution of cells, it does not only matter which metabolites are formed, but also through which reaction sequences they are formed [49,55,56]. Metabolic networks are intrinsically of self-inhibitory nature and struggle with high metabolite concentrations, as many biochemical reactions interfere with each other [57]. This indicates that cells were not exposed to high metabolite concentrations during their evolution and that metabolic networks are selected against redundancy and chemical diversification. As a consequence, it is evolutionarily difficult to replace one reaction sequence with another, even if they produce the same product. Second, it is increasingly recognized that the reaction sequences of metabolism are integrated into the functional principles of biological systems beyond the production of biomolecules [58,59]. For instance, metabolism acts as a sensing and signaling system to dynamically respond and counter stress, to control growth, to determine which molecules are to be transported across the membrane, and to protect cells from damage-causing chemical processes [60–65]. Finally, metabolic networks are of small-world nature, and metabolism can operate far from equilibrium with the environment in which many cells live.

Indeed, there are different possibilities on how the early metabolism was supplied with its substrates, but only one of these scenarios suggests that all life essential metabolites had to accumulate to high levels in the environment before life could start. Clues are provided by the modern metabolic network, in which anabolism and catabolism are highly intertwined. They are driven by a largely overlapping set of biochemical reactions, indicating anabolism and catabolism evolved in parallel rather than one after the other [2,3,66]. In other words, early forms of the metabolic network might very well have formed the life essential metabolites at low concentration but at constant flux. We speculate that low concentration but constant flux reactions might have also been the source of the life essential sugar phosphates that are central to metabolism. In modern cells, the upper glycolytic sugar phosphates are typically formed through the phosphorylation of glucose (or another hexose), or, through the interconversion and condensation of smaller sugar phosphates. In our previous work, we have demonstrated the continuous accumulation of fructose-1,6,-bisphosphate in ice or in rehydration-desiccation cycles, formed from the low-stability 3-carbon phosphates [22]. The work of us and others [36,38] hence shows that the arsenal of nonenzymatic reactions includes pathways that are considered “anabolic” in the modern cell. It has further become clear that a large number of different biomolecules are required to form a functional cellular system; the origin of metabolism was tightly bound to compartmentalization [67].

Recently, increasing evidence has emerged pointing that the structure of the metabolic network could have been directly primed by nonenzymatic metabolic interconversions, in particular those driven by ferrous iron. Fe(II) is believed to have reached up to millimolar concentrations in Archean aquatic environments including the Earth’s early oceans, as the reduced form of iron was stable in the reducing preoxygenation atmosphere and is highly water soluble [43,68,69]. Nonenzymatic chemical interconversion systems driven by Fe(II) resemble the structural organization of the EMP pathway, or glycolysis, the PPP, the oxidative and reduced Krebs cycle, gluconeogenesis and the Wood–Ljungdahl pathway [22,34–38,41,42,44,70].

The motivation for our study was to gain insights into an unsolved question of early metabolic evolution. How could environmentally catalyzed reaction sequences underlie or favor the evolution of enzyme-catalyzed pathway structures? How could the topology of a nonenzymatic reaction sequence become encoded in an enzyme-catalyzed pathway? Previously named the “end product problem,” it is unlikely that multiple metabolic enzymes came into being at once before a selectable product can be formed. However, the metabolic control theory suggests a path out of this dilemma. The performance of any reaction sequences can be improved upon altering the chemistry of the most rate-limiting steps. This situation suggests a gradual stepwise enzymatization of previous nonenzymatic reaction sequences. Once one reaction is improved to the extent it is no longer rate limiting, the selection pressure would shift to the next reaction (Fig 1A). An argument for this model is that it implies that the nonenzymatic reaction sequence would remain imprinted in the structure of the evolved enzyme-catalyzed pathway.

This model allows us to speculate about the point in time when first enzymes were selected. The metabolic network evolved before LUCA; the colonization of different niches required a functional metabolic system. On the other end of the spectrum, the evolution of modern enzymes required an at least rudimentary transcription and translation machinery in place. In turn, the transcription and translation machinery required the metabolites produced by metabolism. We can hence speculate that the rudimentary transcription and translation machinery was supplied by nonenzymatic reactions and that modern enzymes were selected and sequentially populated the nonenzymatic reaction sequences in parallel evolution. The properties of nonenzymatic chemical reactions instead are not subject to Darwinian selection and have hence not changed during the timeline of evolution.

A testable requirement of this model is that simple components that provide the foundation for the selection of enzymes need to be able to interact with the nonenzymatic reaction sequence and, if any, improve their rate-limiting steps. The ability of amino acids to be both enhancers and products of the primordial metabolic network at the same time offers beneficial feedback mechanisms [71,72]. Networks contributing with intermediates that reinforced metal catalysis or reaction stability would have been incorporated into nascent metabolic pathways. Thus, single amino acids (or subsequently, small peptides) enucleating on metal centers would have progressively relegated metals, making metabolism less dependent on environmental catalysts. Eventually this process ends with status quo, in which about 40% of modern enzymes use metal cofactors [73]. In testing this precondition on an iron-driven reaction network of glycolysis and the PPP intermediates, we have discovered that cysteine, a sulfur-containing amino acid, can alter a broad range of activities. It might be possible for these reactions to go through intermediates that one may not find in present day biochemistry. However, in this study, we focus on the direct conversion of 6-phosphogluconate by cysteine interacting with Fe(II), promoting the formation of ribose-5-phosphate, in analogy to a rate-limiting reaction of the oxidative PPP. Although iron is a more effective driver of the reaction, cysteine acts additively on top of the environmental iron concentration, so that the highest reaction rate is reached when iron and cysteine are both present. Moreover, we noted that, specifically at ambient temperatures (20°C), which could have been key to the origin of the metabolic networks, the reaction achieved a high specificity for ribose-5-phosphate formation.

The fact that we observed such properties for cysteine is important for at least 2 reasons. Firstly, cysteine has been previously implicated in the nonenzymatic catalysis of glycolysis and in particular in acetyl-CoA formation, even though such study used large amounts of reagents and remained mainly qualitative [21]. Secondly, organic chemistry struggled for a long time to synthesize cysteine in reaction processes that generated the other amino acids, leading to the speculation that cysteine could be a late product of evolution [74,75]. However, it has recently been reported that cysteine forms in substantial quantities in simple chemical reactions that can have prebiotic pendants [39]. At the same time, cysteine is capable of promoting nonenzymatic peptide ligation reactions in water [39]. The amino acid hence fulfills several conditions that we consider important to act as a primer in the origin of amino acid–based enzymes: (i) it does interact with the environmentally abundant, universal metabolic catalyst, iron; (ii) it can promote chemical reactions, like the formation of an essential component of RNA, ribose-5-phosphate, through chemistries resembling extant cellular metabolism in moderate environmental conditions; and (iii) it could act as a primer for the formation of polypeptides in nonenzymatic ligation reactions. Our results hence propose that cysteine, or, more specifically, iron cysteine complexes, could have been key components in the early selection of enzymatic catalysis.

Regarding the chemical mechanisms, the role of cysteine could be that of a nucleophile as observed in the case of the enzyme aldehyde dehydrogenase [76]. Although the exact chemistry behind the nonoxidative reactions remains to be fully elaborated, the described system is thought to establish the upper intermediates of the PPP and EMP pathways via a series of reactions observed in Maillard reactions as well as thiol-mediated oxidation reactions [77]. In addition, several of the 6-carbon phosphate carbohydrates could have formed recursively from condensation reactions in freeze-thaw cycles under plausible prebiotic conditions [22,78]. Cysteine sulfur condenses with a carbonyl functionality allowing for the facile dehydrogenation of glucose 6-phosphate. The resulting thioester undergoes hydrolysis to give the corresponding carboxylic acid, 6PG. A plausible mechanism for the subsequent conversion from 6PG to R5P has been proposed (S8 Fig), but it will require additional experimental work to validate these predictions. In addition to this observed organocatalysis by cysteine, we also show evidence of interactions between different components—the sugar phosphate, the metal, and cysteine. We propose a ligand–metal interaction between the amino acid and the metal, where the side-chain (thiol) residue, the carboxylic acid, and the amine group of cysteine participate in the coordination with the metal.

It may seem puzzling that the optimum conditions for the reaction occur at mildly acidic pH given that cysteine nucleophilicity would increase with the pH. However, a higher pH also increases the concentration of hydroxyl (OH^−^) in the medium leading to precipitation of the metal catalyst as oxides, in addition to causing a destabilization of cysteine. Furthermore, in the case of the metal-free version of ribose-5-phosphate formation, a higher pH would deplete the availability of free protons that are required for the catalyst. Hence, a mildly acidic media favors solubilized metal species while still retaining the nucleophilicity of the ligands. Given that the isoelectric point of cysteine is close to pH 5, we speculate that the deprotonated carboxylic group could coordinate with Fe(II), in addition to the coordination by the thiol group. From our investigation with different cysteine analogues, we indeed observe that both carboxylic and thiol groups are more critical for reactivity than the amino group.

Our data do not reach to attribute the observed additive effects on the kinetic rates with both metal and amino acid species to the contact interactions in the ternary complex. In fact, we show that both iron and cysteine can drive the reaction independently. It is hence very well plausible that the additive reaction rate when cysteine and iron are combined is explained by both the ternary adduct and the independent action of Fe and Cys on the metabolic transformation. However, the amino acid ligand, in addition to acting as a surrogate of metal catalysis, might also act as a scaffold between the substrate and the metal. This could be regarded as a plausible step in the transition from simple metal–ligand catalyst to longer peptides based on metal centers [79,80]. The metal sites could, in turn, have acted as templates for assembling various combinations of amino acids, favoring reaction and favorable peptide linkages.

In summary, we have shown that the presence of cysteine, a proteinogenic amino acid that can form in simple, prebiotic reactions and can catalyze peptide ligation [39], alters the properties of a nonenzymatic network that resembles central metabolism and promotes the formation of ribose-5-phosphate, the RNA backbone metabolite. Our results hence show that reactions of metal-catalyzed networks can be enhanced, or even replaced, by simple amino acid–based catalysis. Moreover, cysteine and iron form complexes and could have therefore functioned like “minimal enzymes,” allowing a scenario in which nonenzymatic, fuzzy chemical networks serve as a directing template for the formation of early enzymes and, eventually, metabolic pathway structures. This scenario implies that environmental- or metal- driven nonenzymatic reactions contain rate-limiting reactions that can be an amino acid accelerated reaction. This process can provide a solution for the “end product problem,” as these reactions sequences can be improved by altering one step at a time and suggests a scenario in which a stepwise enzymatization of environmental reaction sequences imprinted their structure in the evolving enzyme-catalyzed pathways.

## Materials and methods

### Materials

All chemicals were obtained from Sigma-Aldrich (UK). UPLC/MS grade acetonitrile (AcCN) and water were purchased from Greyhound Chemicals (UK) (see Supporting information Methods in S1 Text for further details).

### Sample preparation for reaction kinetics experiments

Samples consisting of individual sugar phosphates dissolved (in the μM range) in 200 μL aqueous solutions (either in 50 mM phosphate or UPLC/MS grade water) were prepared fresh. These were placed in 2-ml screw thread amber autosampler glass vials (Agilent Technologies, Germany) and sealed in an anaerobic chamber (Coy Laboratory Products, MI, USA) following 3 vacuum/N_2_ injection cycles to remove oxygen content, typically reaching 8 to 12 ppm O_2_ levels. Samples were then incubated in a water bath at the indicated temperature for different times, after which reactions were stopped by immediate transfer to ice. Samples were stored at −20°C until thawed for tandem liquid chromatography–mass spectroscopy (LC–MS/MS) measurements. The incubation time points were specifically set for each metabolite based on its previously reported stability [34,44]; these were typically defined at increasing time intervals and spanned for up to 6 hours in the experiments at 70°C.

### LC–MS/MS and quantification of sugar phosphate interconversions

Samples were analyzed for sugar phosphate content by an LC–MS/MS system, using a triple-quadrupole device (Agilent 6460) operating in SRM mode (parameter specifications and metabolite ion transitions defined as in [34]; see also [81]. A reverse-phase, C8 chromatographic column (Zorbax SB-C8 RRHD 2.1 × 100 mm 1.8 μm from Agilent Technologies) was used for the separation, with 2 binary acetonitrile/water mobile phases (Buffer A: 90% Water, 10% AcCN; Buffer B: 50% Water, 50% AcCN) applied in gradient, both containing 750 mg/l octylammonium acetate as ion-pairing reagent. Details were as followed: 7.5-minute cycle at isocratic 0.6 ml/min flow, with elution with 5% Buffer B for 3.5 minutes, followed by a 2.5-minute ramp to 70% of B, 0.5 minutes at 80% B and 1-minute re-equilibration to 5% B. Individual sugar phosphates could be resolved by retention time as well as fragmentation pattern, as contrasted with external standards. Only Ru5P and X5P (229 -> 97 m/z) and 2PG and 3PG (185 -> 97 m/z) could not be discriminated and were considered together in the analysis. To avoid possible biases in quantification due to long-batch effects (e.g., variations in instrument sensitivity), sample triplicates were randomized in the autosampler positions before MS measurements.

MS/MS data were analyzed with the quantitative MassHunter software (Agilent Technologies), with manual supervision of the automated signal-peak integration. Several replicates of sugar phosphate external-standard dilution series allowed translating signal intensities into absolute concentrations. This was achieved with less accuracy in the case of E4P due to its broad, low-intensity peak, as described in previous work [82] and because of its remarkably poor purity (50%) in the commercially available standard. Also, the lack of a commercial standard for 6PGL forced us to estimate its concentration from the rapid favored equilibrium with 6PG occurring at a strong acidic pH. Concentration time courses were reconstructed with MATLAB. Only cases with over-background product detection showing a congruent increase between triplicates were considered as significant transformations and analyzed for the kinetics. Reaction kinetics were fitted according to one of the following models, depending on the adequacy to the experimental data (R^2^ values): a least squares linear production model, a saturating hyperbolic model or sigmoidal Gompertz function, or a mixed model comprising a first linear phase of maximum (initial) growth rate and a slow exponential decay. Rates, when reported, are shown in terms of the maximum (initial) slopes of the reaction kinetics fits.

### NMR studies of metabolite–iron interaction

Samples containing a final metabolite concentration of 20 mM were prepared in deuterated water (D_2_O) containing 0.05 mM trimethylsilylpropanoic acid (TSP) as internal standard, sodium azide and 50 mM sodium phosphate at a pH 5. FeCl_2_ was added at concentrations of 0, 0.1, 0.2 and 1 mM. NMR experiments were carried out on freshly prepared samples using an AVANCE II+ (Bruker, Rheinstetten, Germany) NMR spectrometer operating at 500.13 MHz for the ^1^H frequency using a 5 mm TXI probe. Basic 1D spectra were collected using a solvent suppression pulse sequence based on a one-dimensional version of the nuclear Overhauser effect spectroscopy (NOESY) pulse sequence to saturate the residual ^1^H water signal (relaxation delay = 2 seconds, t1 increment = 3 μs, mixing time = 150 ms, solvent presaturation applied during the relaxation time and the mixing time). A total of 128 transients were collected into 16 K data points over a spectral width of 12 ppm at 320 K.

Longitudinal relaxation times (*T*_1_) were measured using the Inversion recovery pulse sequence, 180-τ-90, with pulse spacing τ having the following values: 0.01 seconds, 0.02 seconds, 0.03 seconds, 0.05 seconds, 0.1 seconds, 0.25 seconds, 0.5 seconds, 0.75 seconds, 1 seconds, 1.5 seconds, 2 seconds, 4 seconds, 8 seconds, 15 seconds, 20 seconds, and 25 seconds. The pulse repetition time was set at 20 seconds. A total of 8 transients were collected into 16 K data points over a spectral width of 20 ppm at 320 K.

Diffusion coefficients were measured using a bipolar gradient pulse pair with a spoil gradient pulse (stebpgp1s), with 16 incremental steps in the gradient strength linearly ramped from 2% to 95% of the maximum gradient strength. Moreover, 16 scans per increment step were collected into 16K data points over a spectra width of 19 ppm at 320 K. The gradient pulse length was set at 1,000 μs, while the time between pulses at 0.1 seconds. The pulse repetition time was set at 2 seconds.

1D NMR spectra were processed using TopSpin v.3.2 (Bruker, Rheinstetten, Germany). Free induction decays were Fourier transformed following multiplication by a line broadening of 1 Hz, and referenced to TSP at 0.0 ppm. Spectra were automatically phase and baseline corrected. For the relaxation times and diffusion coefficients, data were analyzed using the T1/T2 Relaxation module present in TopSpin. All experiments were carried out at least by triplicate.

## Supporting information

S1 Fig Representative examples of nonenzymatic sugar interconversions modulated by amino acids. Product formation time courses obtained at 70°C from 100 μM substrate in the presence of different subgroups of amino acids (at a total concentration of 400 μM) are shown for different prototypic reactions: diverse transformations accelerated by the sulfur-containing amino acids cysteine and methionine, some of which are negatively affected by at least another group of amino acids (top 3 panel rows); F1,6BP dephosphorylation, enhanced by negatively charged amino acids, with antagonistic effects from positively charged amino acids (fourth set of panels); a pentose-phosphate isomerization, where the role of cysteine and methionine is actually as inhibitors (bottom panel row). Asterisks indicate reaction rates that were significantly different—either higher (green) or lower (red)—than in the aqueous control without amino acids (last column of panels) (Wilcoxon rank sum test, *p* = 0.1). “All mixed” stands for samples containing all 20 amino acids at a total concentration of 400 μM (i.e., 20 μM each). Data points shown in gray (*N* = 3 independent experiments) (see S1 Data).
(TIF)

S2 Fig pH-dependent activity of cysteine on the formation of different products from 6-phosphogluconate. **(A)** Concentrations of sugar phosphate products detected by LC/MS after 6-hour incubation of 100 μM 6PG at 70°C in 50 mM phosphate solution at different pHs, either in the absence (purple lines) or presence (green lines) of 400 μM cysteine. The optimum pH range where the reaction enhancement by cysteine was larger is highlighted as a shaded, gray region where applicable. A control consisting of 100 μM 6PG in unbuffered (aqueous) conditions, as well as a negative control without 6PG are shown for comparison. Data shown as mean ± SD (*N* ≥ 3 in all conditions) (see S1 Data). **(B)** Same protocol was followed but in the presence of a metal ion, 200 μM FeCl_2_, either without (purple lines) or with 400 μM cysteine co-present (green lines). Data shown as mean ± SD (*N* ≥ 3 in all conditions) (see S1 Data and Supporting information Note in S1 Text). 6PG, 6-phosphogluconic acid; LC/MS, liquid chromatography–mass spectrometry.
(TIF)

S3 Fig Stability of cysteine at 70°C under different experimental conditions. **(A)** Time evolution of cysteine concentration during incubation in 50 mM phosphate solution at different pHs. Lines correspond with exponential decay fits. Corresponding rates are shown in **(B)**: median ± IQR (*N* ≥ 3) (see S1 Data). **(C)** For those conditions of intermediate pH, cysteine time courses were reanalyzed both in absence and presence of increasing concentrations of the substrate, 6PG, with just very slight changes detected. Corresponding rates are shown in **(D)**: median ± IQR (*N* ≥ 3) (see S1 Data). In all experiments, cysteine concentration was quantified spectrophotometrically using Ellman’s reagent (see Materials and methods and Supporting information Methods in S1 Text). 6PG, 6-phosphogluconate.
(TIF)

S4 Fig Ribose-5-phosphate yields obtained from 6PG with different cysteine analogues. A total of 800 μM 6PG was incubated at 70°C in a 50 mM phosphate solution pH 5 containing 400 μM of cysteine analogue. **(A)** Representative chromatograms obtained by LC–SRM after 6-hour incubation under control conditions without amino acids (−), or in the presence of serine or cysteine, showing how serine, unlike cysteine, has no significant effect on product formation versus control conditions (*N* = 3 independent experiments) (see S1 Data). **(B)** The concentration of R5P formed after 6 hours is shown—relative to the 400 μM cysteine condition—for isomers or close homologues bearing the same functional groups (i.e., D-cysteine, DL-homocysteine and reduced glutathione (GSH)), structural analogues differing in one or several functional groups (3-mercaptopropionic acid, cysteamine, β-mercaptoethanol and L-serine), and oxidized cysteine derivatives (L-cystine, cysteine sulfonic acid and oxidized glutathione (GSSG)), evidencing the importance of the thiol group. Error bars represent mean ± SD (*N* = 3) (see S1 Data and Supporting information Note in S1 Text). 6PG, 6-phosphogluconate; LC–SRM, liquid chromatography–selective reaction monitoring; R5P, ribose-5-phosphate.
(TIF)

S5 Fig Ribose-5-phosphate formation rates with different molar ratios of amino acid and metal. A total of 800 μM 6PG was incubated at 70°C in 50 mM phosphate solution pH 5 containing different molar ratios of cysteine and FeCl_2_ but at the same total concentration, 150 μM. **(A)** Detailed time courses in R5P formation are shown. Lines represent best hyperbolic fits. *N* = 3 per condition (see S1 Data). **(B)** Estimated initial rates (error bars: mean ± SD) are plotted as a function of the ratio between both additives (see S1 Data). The gray dashed line represents the null-model expectation of weighted additive contributions with no patent inter-species dynamic interaction. 6PG, 6-phosphogluconate; R5P, ribose-5-phosphate.
(TIF)

S6 Fig ^1^H-NMR spectra of nonreactive substrate or amino acid substituents and interactions with paramagnetic Fe^2+^. Fe^2+^ broadens the proton signal peaks of closely proximal molecules in solution (in this case solvent is 50 mM phosphate pH 5 in D_2_O). **(A)** Chemical structures of glucose 6-phosphate (G6P) and methionine, with positions of resonant protons labeled. **(B)** Representative ^1^H-NMR spectra of solutions containing 20 mM G6P + 20 mM cysteine or 20 mM 6PG + 20 mM methionine were compared with those of a solution with the key components of the reaction: 20 mM 6PG + 20 mM cysteine. Addition of 1 mM FeCl_2_ (spectra in red) just slightly affected the individual species when analyzed separately, but extensively distorted the peaks of 6PG and cysteine when these were combined in the same solution, in contrast to the other 2 cases with either G6P or methionine where peak definition remained almost unaltered. This suggests a fairly specific cysteine–6PG interaction that makes them more likely to Fe^2+^ binding. Peak numbers correspond with proton labels in (A). In all cases, representative examples are shown from at least *N* = 3 independent experiments (for visual comparison, spectra are shown normalized to the maximum peak intensity in each case) (see S1 Data). 6PG, 6-phosphogluconate.
(TIF)

S7 Fig Thermal-dependent product specificity over time. A total of 800 μM 6PG was incubated at different temperatures in 50 mM phosphate solution pH 5 containing 75 μM cysteine and 75 μM FeCl_2_, and the formation of different sugar phosphate products was monitored over time. Shown is the cumulative product concentration normalized to the total product quantified at each time by targeted LC/MS. As temperature decreases, the loss of specificity in R5P formation detected at higher temperatures ceases and R5P becomes the predominant sugar phosphate product, also at long term (70% to 80% of total product; blue area). Time ranges are the same as in main Fig 4A and were defined based on the kinetics scaling with temperature. Error bars account for mean ± SD from *N* = 3 independent experiments (see S1 Data). 6PG, 6-phosphogluconate; LC/MS, liquid chromatography–mass spectrometry; R5P, ribose-5-phosphate.
(TIF)

S8 Fig Proposed mechanism for the conversion of 6PG to R5P. Oxidant could be an electron acceptor such as oxygen or Fe(III) generated *in situ*. X could be a lewis acid like Fe2+ or protonated amino group from the amino acid. The pentose phosphate sugar product could be either D-Ribose-5-phosphate or D-Arabinose 5-phosphate, which are diastereomers and cannot be distinguished by LC/MS. 6PG, 6-phosphogluconate; LC/MS, liquid chromatography–mass spectrometry; R5P, ribose-5-phosphate.
(TIF)

S1 Table Effect of oxygen on the formation of ribose 5 phosphate. (PDF)

S1 Data Numerical values underlying summary data displayed in figures. The data for experiments summarized in the figures are included in a single Excel file as individual worksheets for panels in Figs 1D, 1E, 1F, 2B, 2C, 3B, 3C, 3D, 4A, 4B and 4C, as well as S1, S2A, S2B, S3A, S3B, S3C, S3D, S4A, S4B, S5A, S5B, S6B, and S7 Figs.
(XLSX)

S2 Data Sugar phosphate interconversion rates at 70°C in the presence of different amino acids. Summary of nonenzymatic interconversion rates obtained by LC–SRM and models used for fitting experimental data. See individual rates in S1 Data. LC–SRM, liquid chromatography–selective reaction monitoring.
(XLSX)

S1 Text Supporting information methods, notes on experimental results, and Supporting information tables. Details of materials and reagents used and the cysteine quantitation procedure are described as Supporting information methods, followed by notes discussing pH-dependent product formation and the role of cysteine functional groups, and Supporting information tables with additional data on trace Fe quantitation and ^1^H-NMR *T*_1_ measurements across proton peaks in different setups.
(PDF)

## Abbreviations

3PG, 3: phosphoglycerate
6PG, 6: phosphogluconic acid
DOSY: diffusion ordered spectroscopy
EMP: Embden–Meyerhof–Parnas
LC–MS/MS: tandem liquid chromatography–mass spectrometry
LC–SRM: liquid chromatography–selective reaction monitoring
NOESY: nuclear Overhauser effect spectroscopy
PPP: pentose phosphate pathway
TSP: trimethylsilylpropanoic acid
