# Supplementary figures and images for: Cysteine and iron accelerate the formation of ribose-5-phosphate, providing insights into the evolutionary origins of the metabolic network structure
Source: PLoS Biol. 2021 Dec 3;19(12):e3001468. doi: 10.1371/journal.pbio.3001468 (PMC8673631; doi:10.1371/journal.pbio.3001468)

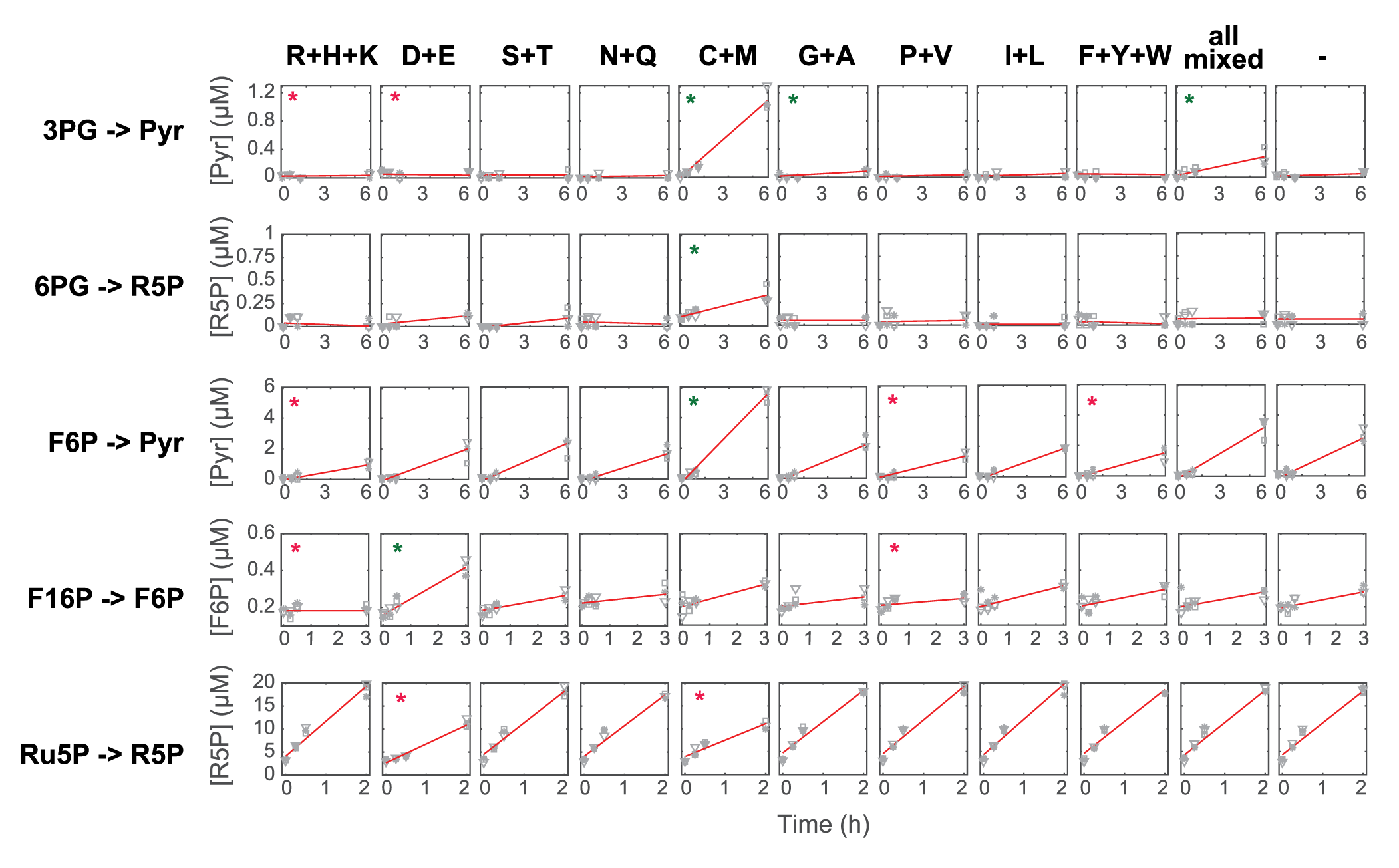

Supplement: S1 Fig — Product formation time courses obtained at 70°C from 100 μM substrate in the presence of different subgroups of amino acids (at a total concentration of 400 μM) are shown for different prototypic reactions: diverse transformations accelerated by the sulfur-containing amino acids cysteine and methionine, some of which are negatively affected by at least another group of amino acids (top 3 panel rows); F1,6BP dephosphorylation, enhanced by negatively charged amino acids, with antagonistic effects from positively charged amino acids (fourth set of panels); a pentose-phosphate isomerization, where the role of cysteine and methionine is actually as inhibitors (bottom panel row). Asterisks indicate reaction rates that were significantly different—either higher (green) or lower (red)—than in the aqueous control without amino acids (last column of panels) (Wilcoxon rank sum test, p = 0.1). “All mixed” stands for samples containing all 20 amino acids at a total concentration of 400 μM (i.e., 20 μM each). Data points shown in gray (N = 3 independent experiments) (see S1 Data). (TIF) [file pbio.3001468.s001.tif]

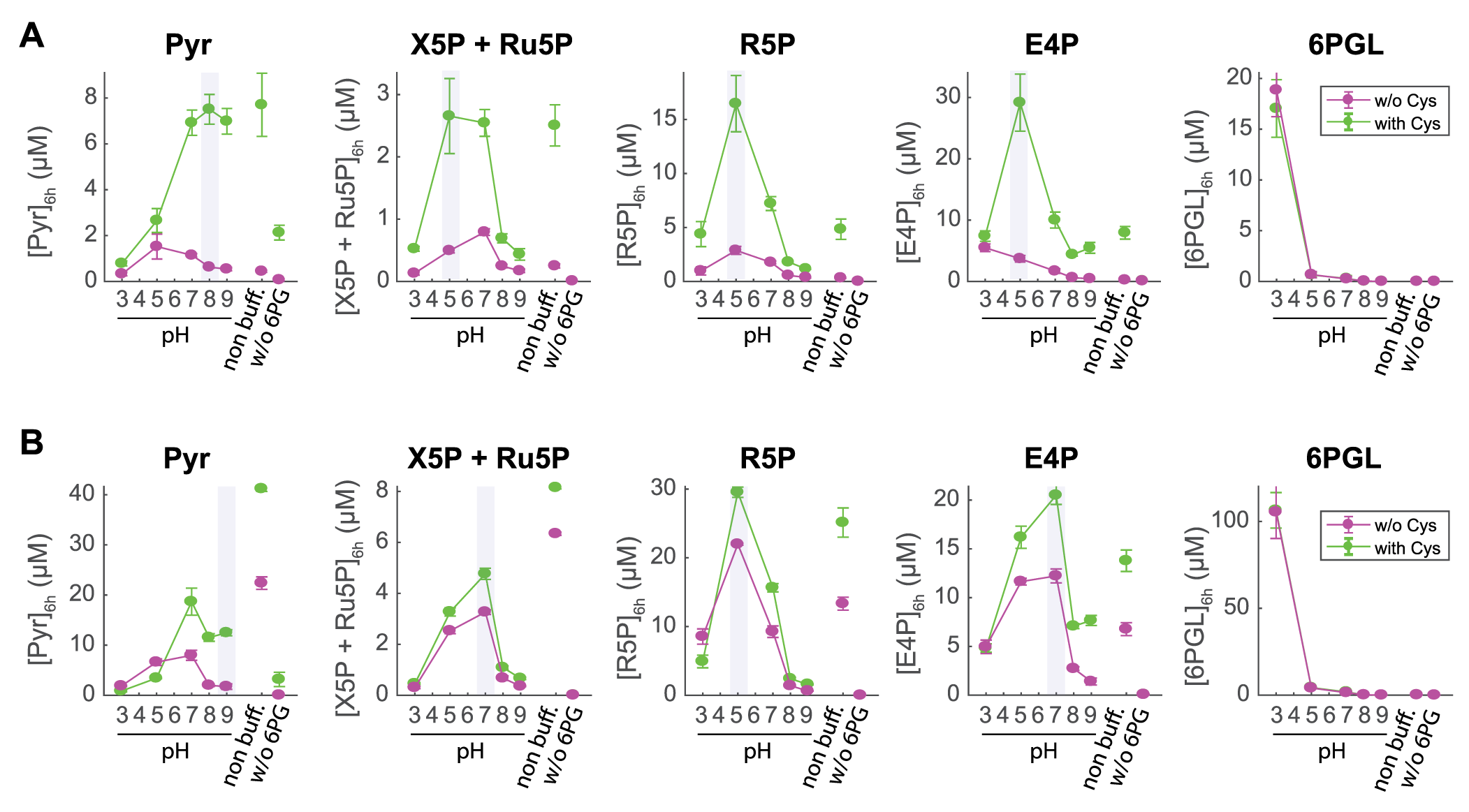

Supplement: S2 Fig — (A) Concentrations of sugar phosphate products detected by LC/MS after 6-hour incubation of 100 μM 6PG at 70°C in 50 mM phosphate solution at different pHs, either in the absence (purple lines) or presence (green lines) of 400 μM cysteine. The optimum pH range where the reaction enhancement by cysteine was larger is highlighted as a shaded, gray region where applicable. A control consisting of 100 μM 6PG in unbuffered (aqueous) conditions, as well as a negative control without 6PG are shown for comparison. Data shown as mean ± SD (N ≥ 3 in all conditions) (see S1 Data). (B) Same protocol was followed but in the presence of a metal ion, 200 μM FeCl2, either without (purple lines) or with 400 μM cysteine co-present (green lines). Data shown as mean ± SD (N ≥ 3 in all conditions) (see S1 Data and Supporting information Note in S1 Text). 6PG, 6-phosphogluconic acid; LC/MS, liquid chromatography–mass spectrometry. (TIF) [file pbio.3001468.s002.tif]

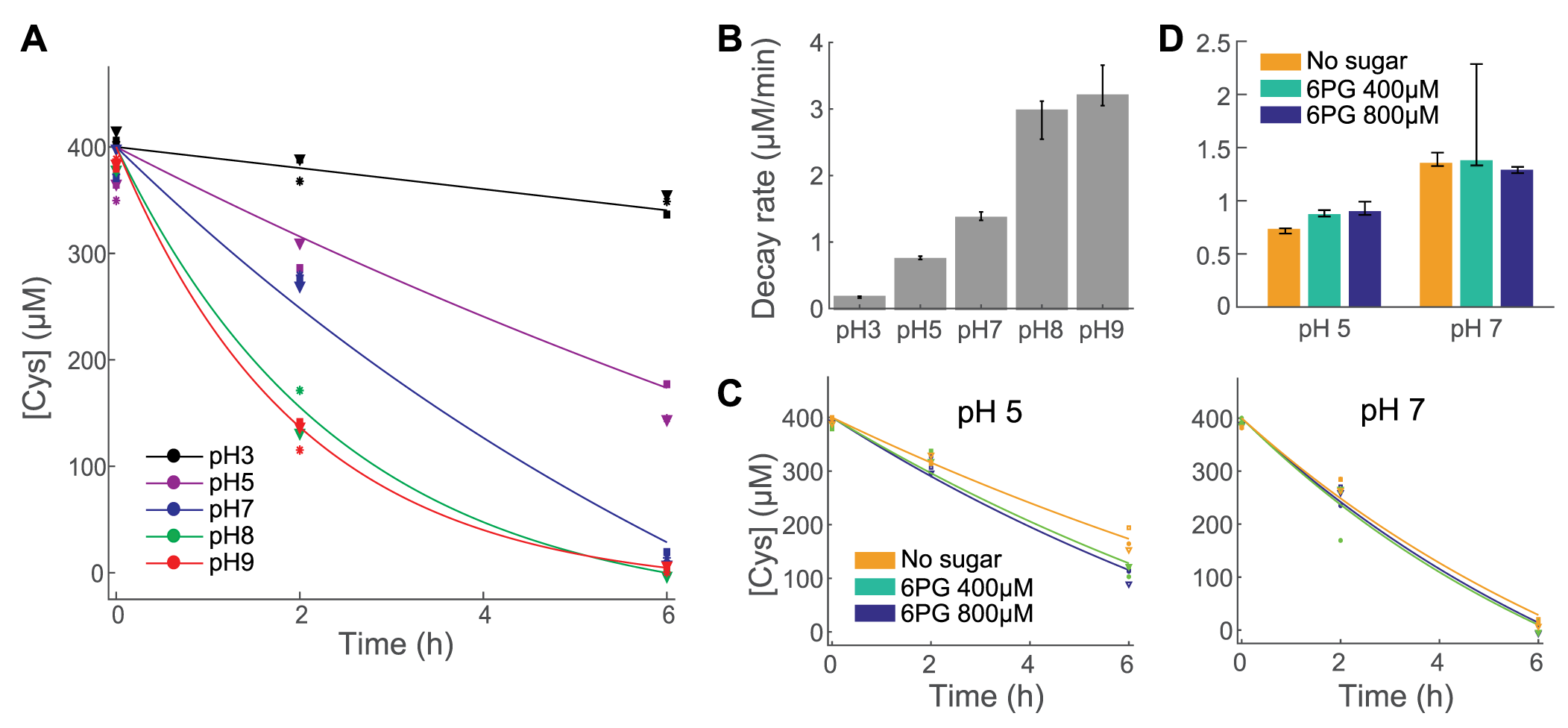

Supplement: S3 Fig — (A) Time evolution of cysteine concentration during incubation in 50 mM phosphate solution at different pHs. Lines correspond with exponential decay fits. Corresponding rates are shown in (B): median ± IQR (N ≥ 3) (see S1 Data). (C) For those conditions of intermediate pH, cysteine time courses were reanalyzed both in absence and presence of increasing concentrations of the substrate, 6PG, with just very slight changes detected. Corresponding rates are shown in (D): median ± IQR (N ≥ 3) (see S1 Data). In all experiments, cysteine concentration was quantified spectrophotometrically using Ellman’s reagent (see Materials and methods and Supporting information Methods in S1 Text). 6PG, 6-phosphogluconate. (TIF) [file pbio.3001468.s003.tif]

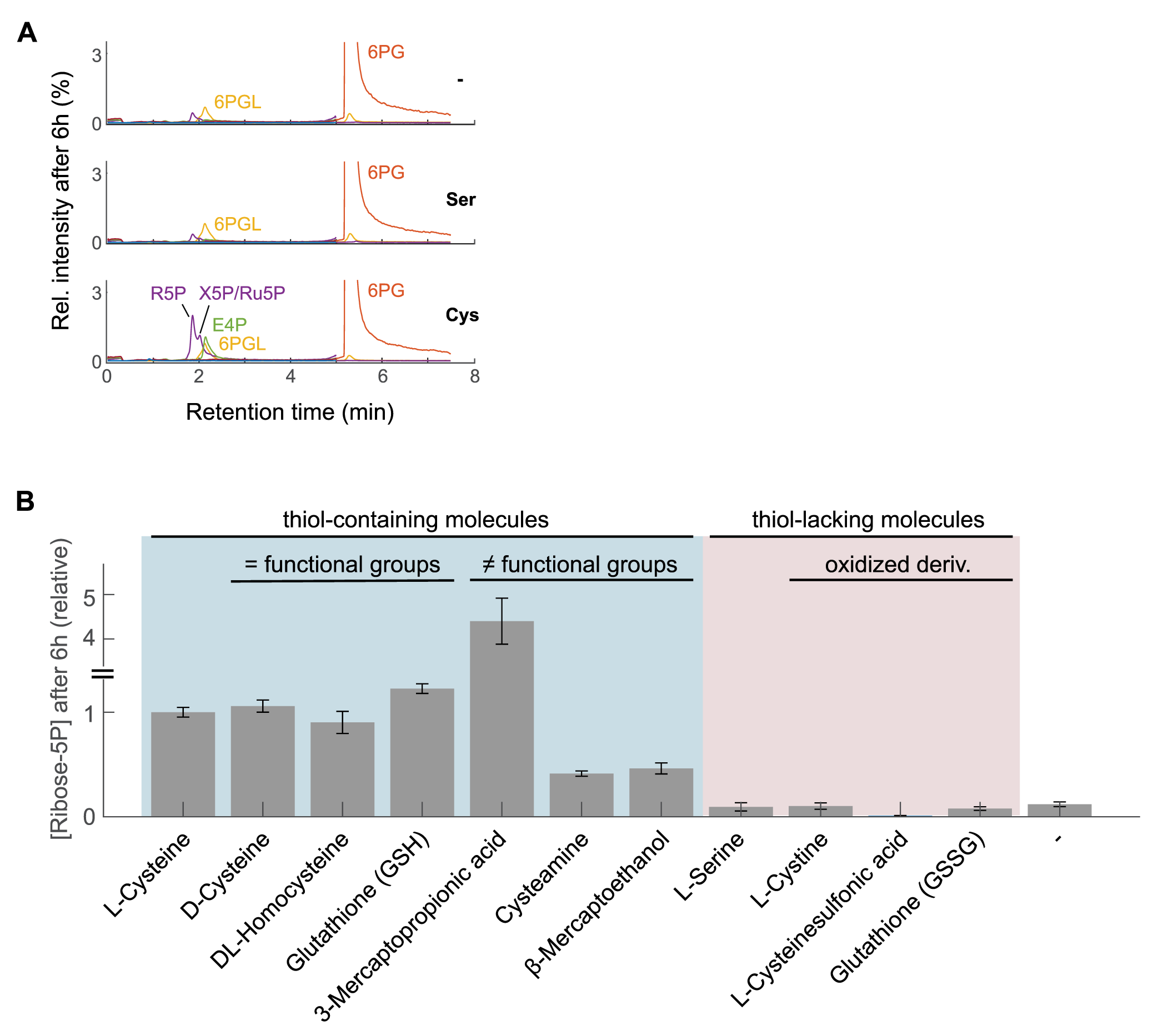

Supplement: S4 Fig — A total of 800 μM 6PG was incubated at 70°C in a 50 mM phosphate solution pH 5 containing 400 μM of cysteine analogue. (A) Representative chromatograms obtained by LC–SRM after 6-hour incubation under control conditions without amino acids (−), or in the presence of serine or cysteine, showing how serine, unlike cysteine, has no significant effect on product formation versus control conditions (N = 3 independent experiments) (see S1 Data). (B) The concentration of R5P formed after 6 hours is shown—relative to the 400 μM cysteine condition—for isomers or close homologues bearing the same functional groups (i.e., D-cysteine, DL-homocysteine and reduced glutathione (GSH)), structural analogues differing in one or several functional groups (3-mercaptopropionic acid, cysteamine, β-mercaptoethanol and L-serine), and oxidized cysteine derivatives (L-cystine, cysteine sulfonic acid and oxidized glutathione (GSSG)), evidencing the importance of the thiol group. Error bars represent mean ± SD (N = 3) (see S1 Data and Supporting information Note in S1 Text). 6PG, 6-phosphogluconate; LC–SRM, liquid chromatography–selective reaction monitoring; R5P, ribose-5-phosphate. (TIF) [file pbio.3001468.s004.tif]

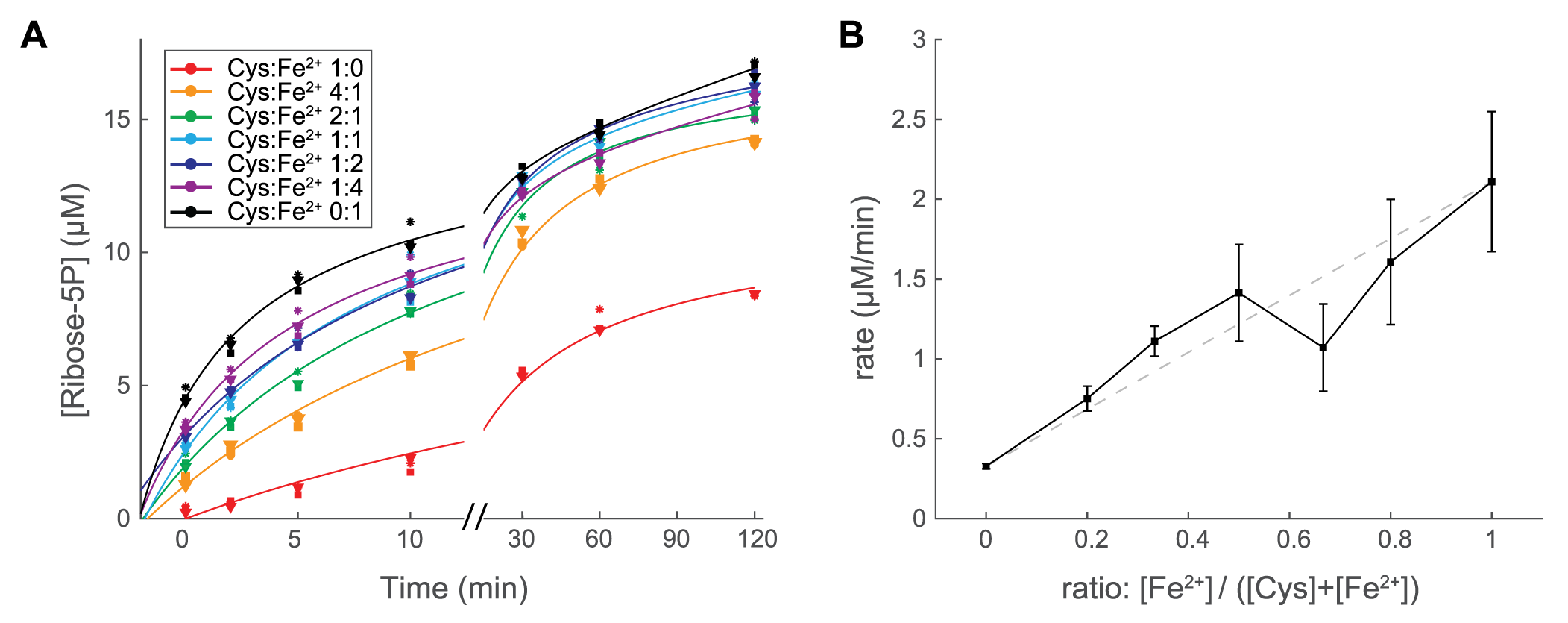

Supplement: S5 Fig — A total of 800 μM 6PG was incubated at 70°C in 50 mM phosphate solution pH 5 containing different molar ratios of cysteine and FeCl2 but at the same total concentration, 150 μM. (A) Detailed time courses in R5P formation are shown. Lines represent best hyperbolic fits. N = 3 per condition (see S1 Data). (B) Estimated initial rates (error bars: mean ± SD) are plotted as a function of the ratio between both additives (see S1 Data). The gray dashed line represents the null-model expectation of weighted additive contributions with no patent inter-species dynamic interaction. 6PG, 6-phosphogluconate; R5P, ribose-5-phosphate. (TIF) [file pbio.3001468.s005.tif]

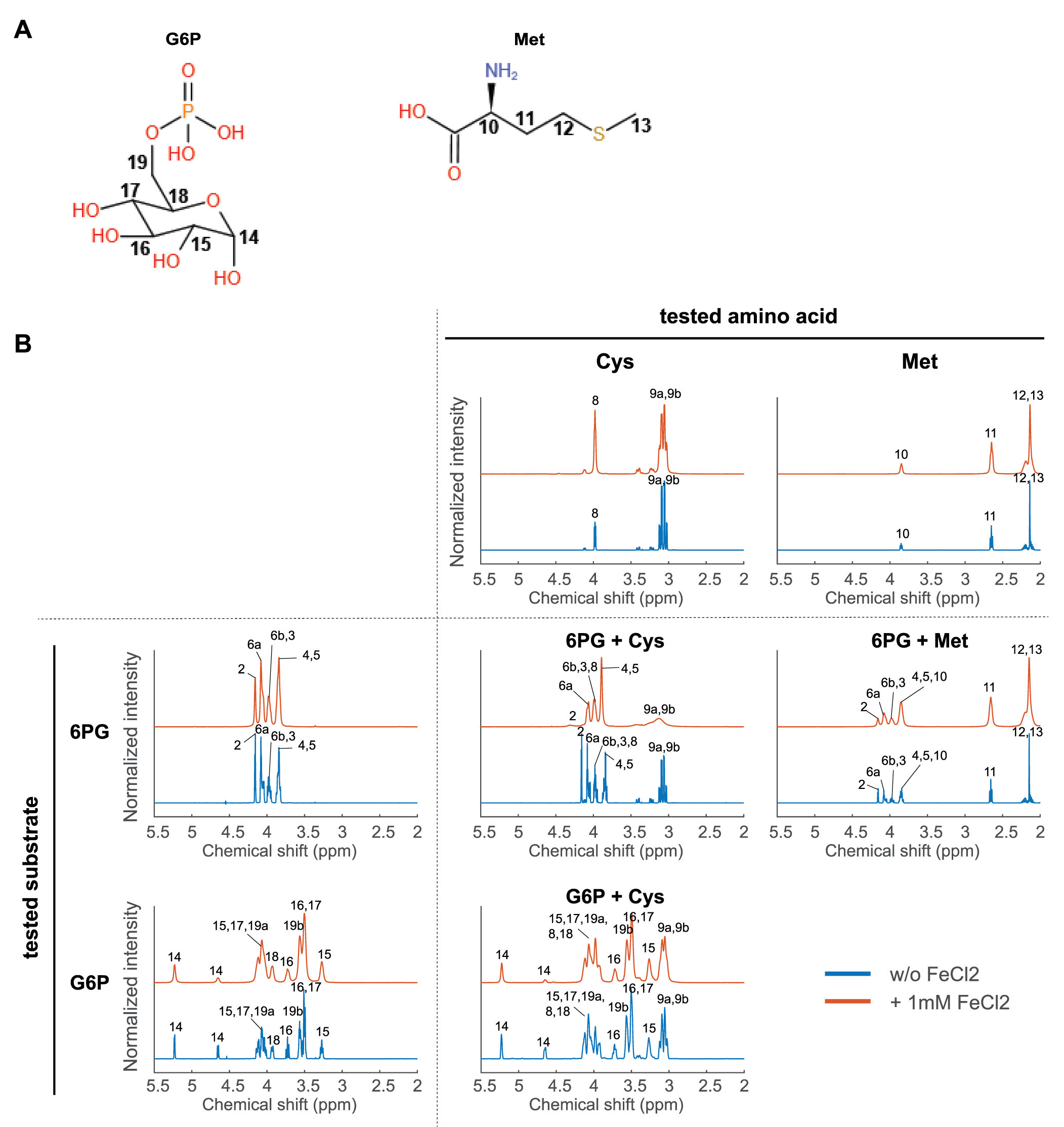

Supplement: S6 Fig — Fe2+ broadens the proton signal peaks of closely proximal molecules in solution (in this case solvent is 50 mM phosphate pH 5 in D2O). (A) Chemical structures of glucose 6-phosphate (G6P) and methionine, with positions of resonant protons labeled. (B) Representative 1H-NMR spectra of solutions containing 20 mM G6P + 20 mM cysteine or 20 mM 6PG + 20 mM methionine were compared with those of a solution with the key components of the reaction: 20 mM 6PG + 20 mM cysteine. Addition of 1 mM FeCl2 (spectra in red) just slightly affected the individual species when analyzed separately, but extensively distorted the peaks of 6PG and cysteine when these were combined in the same solution, in contrast to the other 2 cases with either G6P or methionine where peak definition remained almost unaltered. This suggests a fairly specific cysteine–6PG interaction that makes them more likely to Fe2+ binding. Peak numbers correspond with proton labels in (A). In all cases, representative examples are shown from at least N = 3 independent experiments (for visual comparison, spectra are shown normalized to the maximum peak intensity in each case) (see S1 Data). 6PG, 6-phosphogluconate. (TIF) [file pbio.3001468.s006.tif]

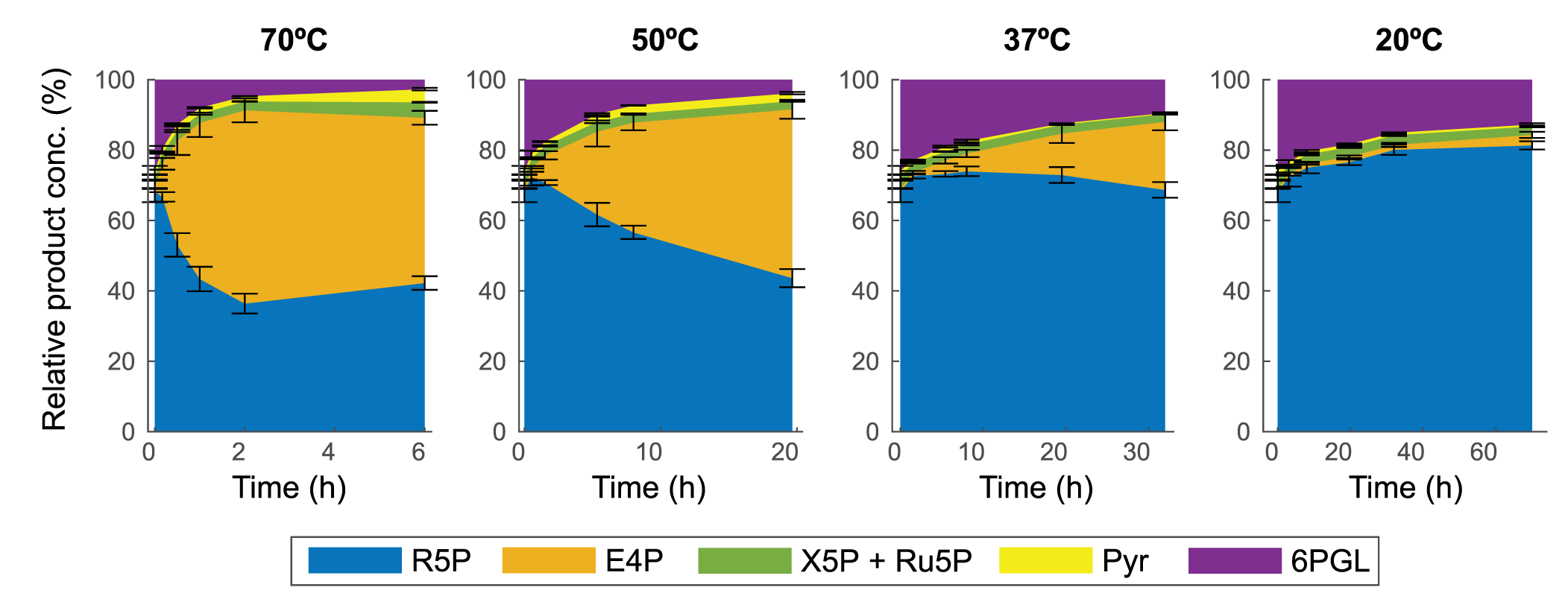

Supplement: S7 Fig — A total of 800 μM 6PG was incubated at different temperatures in 50 mM phosphate solution pH 5 containing 75 μM cysteine and 75 μM FeCl2, and the formation of different sugar phosphate products was monitored over time. Shown is the cumulative product concentration normalized to the total product quantified at each time by targeted LC/MS. As temperature decreases, the loss of specificity in R5P formation detected at higher temperatures ceases and R5P becomes the predominant sugar phosphate product, also at long term (70% to 80% of total product; blue area). Time ranges are the same as in main Fig 4A and were defined based on the kinetics scaling with temperature. Error bars account for mean ± SD from N = 3 independent experiments (see S1 Data). 6PG, 6-phosphogluconate; LC/MS, liquid chromatography–mass spectrometry; R5P, ribose-5-phosphate. (TIF) [file pbio.3001468.s007.tif]

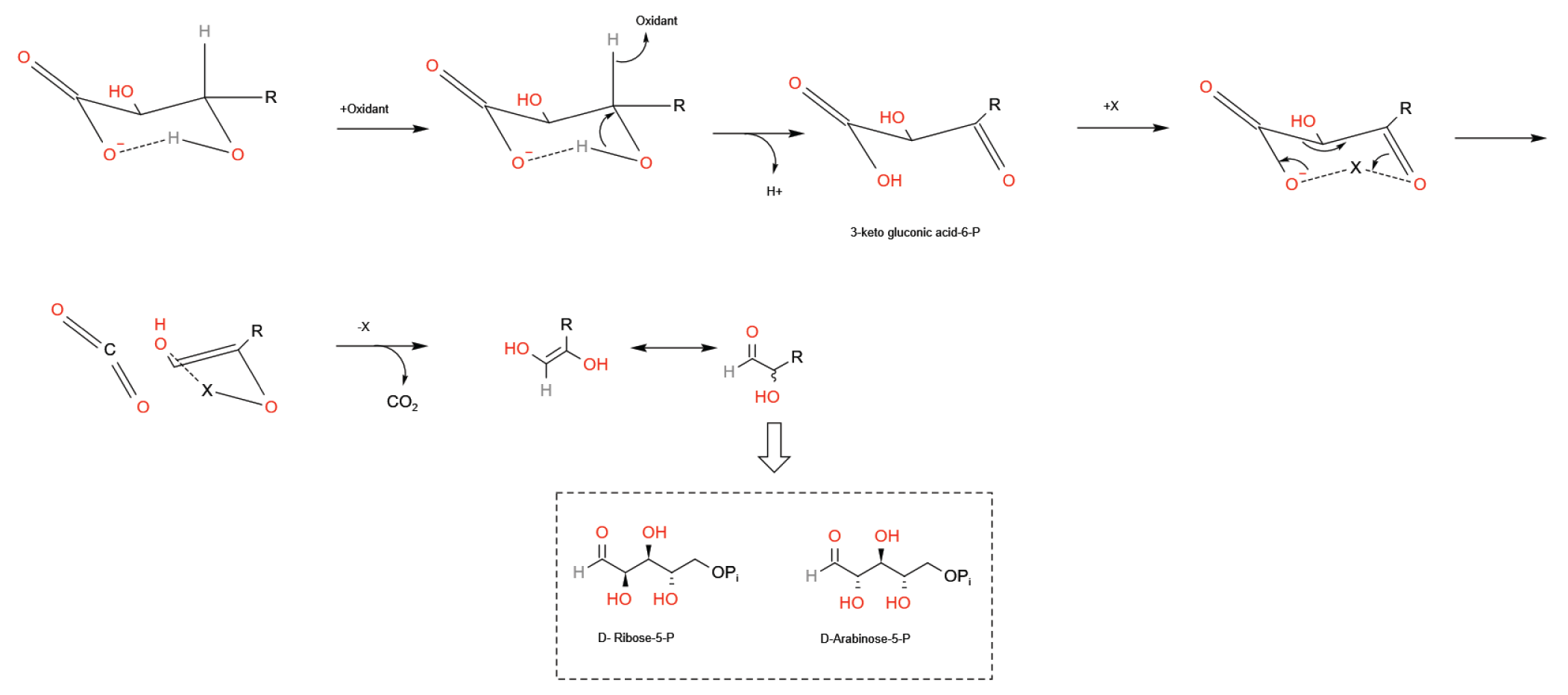

Supplement: S8 Fig — Oxidant could be an electron acceptor such as oxygen or Fe(III) generated in situ. X could be a lewis acid like Fe2+ or protonated amino group from the amino acid. The pentose phosphate sugar product could be either D-Ribose-5-phosphate or D-Arabinose 5-phosphate, which are diastereomers and cannot be distinguished by LC/MS. 6PG, 6-phosphogluconate; LC/MS, liquid chromatography–mass spectrometry; R5P, ribose-5-phosphate. (TIF) [file pbio.3001468.s008.tif]
