# Supplementary material for: Cysteine and iron accelerate the formation of ribose-5-phosphate, providing insights into the evolutionary origins of the metabolic network structure
Source: PLoS Biol. 2021 Dec 3;19(12):e3001468. doi: 10.1371/journal.pbio.3001468 (PMC8673631; doi:10.1371/journal.pbio.3001468)
Supplement: S1 Table — (PDF) [file pbio.3001468.s009.pdf]

S1 Table. Effect of oxygen on the formation of ribose 5 phosphate.

Sl	Reagents*	Ratio of R5P concentration formed under aerobic vs. anaerobic environment
1	400 µM 6PG, 150µM Fe^{2+} and 150µM Cys	3.05
2	400 µM 6PG and 150µM Cys	1.11
3	400 µM 6PG and 150µM Fe^{2+}	2.5
4	400 µM 6PG alone	1

* Reagents were dissolved in phosphate solution 50 mM pH 5.0. The reaction mixture was heated at 70 ºC for 3h under respective environments (ambient or anaerobic chamber). 6PG: 6-phosphogluconate, R5P: ribose 5-phosphate.
