# Supplementary material for: Cysteine and iron accelerate the formation of ribose-5-phosphate, providing insights into the evolutionary origins of the metabolic network structure
Source: PLoS Biol. 2021 Dec 3;19(12):e3001468. doi: 10.1371/journal.pbio.3001468 (PMC8673631; doi:10.1371/journal.pbio.3001468)
Supplement: S1 Text — Details of materials and reagents used and the cysteine quantitation procedure are described as Supporting information methods, followed by notes discussing pH-dependent product formation and the role of cysteine functional groups, and Supporting information tables with additional data on trace Fe quantitation and 1H-NMR T1 measurements across proton peaks in different setups. (PDF) [file pbio.3001468.s012.pdf]

Supporting Information: S1 Text

Cysteine and iron accelerate the formation of ribose-5-phosphate, providing insights into the evolutionary origins of the metabolic network structure

Gabriel Piedrafita[1,†], Sreejith Varma[2], Cecilia Castro[1], Christoph Messner[3], Lukasz Szyrwiel[3], Julian Griffin[1,4] and Markus Ralser[1,2,3,*]

[1] Department of Biochemistry and Cambridge Systems Biology Centre, University of Cambridge, UK.
[2] Department of Biochemistry, Charité University Medicine, Berlin, Germany.
[3] The Molecular Biology of Metabolism Laboratory, The Francis Crick Institute, UK.
[4] Section of Biomolecular Medicine, Department of Metabolism, Digestion and Reproduction, Imperial College London, The Sir Alexander Fleming Building, Exhibition Road, South Kensington, London, SW7 2AZ, UK.

† present address: Spanish National Cancer Research Centre (CNIO), Madrid, Spain.
* markus.ralser@crick.ac.uk

This 'S1 Text' file includes:

Supplementary Methods

Supplementary Note

Tables A-H

References

Supplementary Methods

Materials. All chemicals were obtained from Sigma-Aldrich. ULC/MS grade acetonitrile (AcCN) and water were purchased from Greyhound Chemicals. The anaerobic experiments from the oxygen effect studies were carried out in a glove box (model B; COY Laboratory Products) under an atmosphere of 95% N_2/5% H_2(Air-Liquide GmbH). All the solutions used were degassed in a stoppered bottle/tube with a screw cap with butyl rubber septum by successive cycles of evacuating and N_2 gas flushing at a Schlenk line. The content of ^{56}Fe was measured using an Agilent 7900 ICP-MS instrument fitted with Ni cones and a MicroMistnebulizer in H_2 mode.

Cysteine quantitation. Cysteine was prepared at 400 µM in 50 mM phosphate solutions at the indicated pHs, and incubated at 70°C for different times in anaerobic conditions (typically reaching 8-12 ppm O_2 levels), following the same protocol described for sugar phosphates (Materials and Methods). Sample vials were cooled by transference to ice (but avoiding freezing) and thereafter cysteine levels quantified by colorimetric assay using Ellman's reagent (1), a free sulfhydryl-reactive chemical yielding a measurable yellow product: A stock solution of 5,5'-dithiobis-(2-nitrobenzoic acid) (DTNB) was added to the samples, to a final concentration of 70 µg/mL. Upon short vortexing and 10-min incubation at room temperature, samples were transferred into 96-well plates and the absorbance at 412 nm measured spectrophotometrically (Infinite 200 PRO microplate reader from Tecan). A standard dilution series made from freshly prepared solutions of known cysteine concentrations was used to obtain absolute concentrations.

Supplementary Note

pH-dependent product formation of cysteine-driven 6-phosphogluconate reactions:

Since different products apart from ribose 5-phosphate were accessible from 6-phosphogluconate in (unbuffered) aqueous conditions at 70°C, we inquired how pH changes could affect the reaction specificity and whether the pH would allow tuning the rate efficiency and cysteine preference to form one product over other. Here we comment on the optimal pH for reactivity, and address whether the pH optimum is reaction or product-specific (**S2 Fig**). Samples containing 800 µM 6-phosphogluconate and 400 µM cysteine were prepared in 50 mM phosphate solutions spanning a wide range of pH values (between 3 and 9). Product yields after 6 h incubation at 70°C turned out to be very dependent on pH (**S2 Fig**). While pyruvate was preferentially formed at alkaline pHs, intermediate or mild acidic conditions (around pH 5) were the most favorable for the formation of higher-order sugar species from the PPP, i.e. 5-carbon sugar phosphates, and predominantly ribose 5-phosphate. Additionally, other PPP intermediates not found in the original unbuffered aqueous conditions were detected at mild and strong acidic pHs: erythrose 4-phosphate and 6-phosphogluconolactone, respectively.

Altogether, although product-specific, pH-dependent reaction rate profiles with cysteine were mostly different than those obtained in control conditions (without cysteine) and resulted much more prominent, with relatively narrow optimal pH values, suggestive of catalysis (**S2 Fig**). Only in the case of 6-phosphogluconolactone, production yields were independent of cysteine (**S2 Fig**) – indeed, the formation of this species at pH 3 is consistent with favored proton-driven dehydration of 6-phosphogluconate under acidic conditions. Now, the distinguishable modes of response to pH obtained for the different products may point to alternative driven reactions pathways, but could also result from multi-step sequential chemistry. In this context, we chose to focus the analysis on ribose 5-phosphate (and the other -- less abundant -- pentose phosphates) due to their particular relevance for being the metabolites most immediately related to 6-phosphogluconate (at least in vivo), and thus more likely as primary products of catalysis, as for showing an optimum formation rate around pH 5, far from alkaline conditions where cysteine was found more reactive (**S3 Fig**).

Role of cysteine functional groups in product formation rate:

In order to study the role of cysteine functional groups in the sugar conversion performance we design a time-course experiment with a battery of cysteine analogues (**S4 Fig**). 6-phosphogluconate-derived ribose 5-phosphate formation was analyzed in the presence of each of the following molecular analogues of cysteine (again, at a concentration of 400 µM): Isomers or close homologues bearing the same functional groups (D-cysteine, DL-homocysteine and reduced glutathione (GSH)), structural analogues differing in one or several functional groups (L-serine, cysteamine, β-mercaptoethanol and 3-mercaptopropionic acid), and oxidized cysteine derivatives (cystine, oxidized glutathione (GSSG) and cysteine sulfonic acid) were tested. The results demonstrated that the thiol group (R-SH) was crucial for cysteine activity (**S4 Fig**). No significant formation of ribose 5-phosphate was detected with the alcohol analogue L-serine, nor

with the disulfide-based compounds after 6h incubation with 800 μM 6-phosphogluconate. Conversely, irrespective of slight structural differences, all the thiol-containing analogues mimicked cysteine effects. Interestingly, however, the presence of the carboxylic group was a second feature of advantage, with cysteamine and β-mercaptoethanol showing only modest enhancement effects (**S4 Fig**). In addition, the enhanced reaction rate with 3-mercaptopropionic acid would suggest that the local environment of the carboxylic group is also important. These results point to a relatively specific and complex set of conditions required for catalysis even in a small molecule such as an amino acid.

Table A. Measurement of trace Fe contamination in the reagents used.

Component	Concentration ± RSD (µM)
Cysteine	1.065 ± 0.005
6-phosphogluconate	0.999 ± 0.007
Phosphate solution	0.938 ± 0.011
Cys+6PG under standard reaction conditions*	1.108 ± 0.011

*Dissolved in 50 mM phosphate solution at pH 5.0 and incubated in amber vials at 70 ºC for 3h.

Table B. T_1 relaxation time of cysteine solutions with increasing concentrations of Fe(II).

Fe ratio	3.97 ppm	3.06 ppm
0	10.82 ± 0.77	3.10 ± 0.17
0.005	6.11 ± 0.20	2.63 ± 0.01
0.01	4.54 ± 0.08	2.56 ± 0.03
0.05	3.75 ± 0.34	2.52 ± 0.20

Table C. T_1 relaxation time of 6PG solutions with increasing concentrations of Fe(II).

Fe ratio	4.12 ppm	4.09 ppm	3.96 ppm	3.84 ppm
0	2.74 ± 0.13	2.07 ± 0.06	0.98 ± 0.01	2.23 ± 0.01
0.005	1.35 ± 0.05	1.24 ± 0.06	0.74 ± 0.02	1.31 ± 0.03
0.01	1.21 ± 0.05	1.12 ± 0.04	0.71 ± 0.04	1.19 ± 0.03
0.05	0.89 ± 0.06	0.85 ± 0.04	0.58 ± 0.05	0.97 ± 0.03

Table D. T_1 relaxation time of mixed cysteine-6PG solutions with increasing concentrations of Fe(II).

Fe ratio	4.17 ppm	4.08 ppm	4.00 ppm	3.85 ppm	3.10 ppm
0	3.24 ± 0.31	2.19 ± 0.09		2.28 ± 0.12	2.51 ± 0.21
0.005	0.97 ± 0.03	1.42 ± 0.03	1.10 ± 0.02	1.30 ± 0.02	1.60 ± 0.02
0.01	0.69 ± 0.05	1.02 ± 0.06	0.74 ± 0.02	0.94 ± 0.05	1.20 ± 0.03
0.05		0.46 ± 0.08	0.35 ± 0.04	0.53 ± 0.04	0.49 ± 0.05

Table E. T_1 relaxation time of methionine solutions with increasing concentrations of Fe(II).

Fe ratio	3.85 ppm	2.63 ppm	2.16 ppm
0	6.55 ± 0.10	2.51 ± 0.03	3.34 ± 0.03
0.005	4.65 ± 0.33	2.32 ± 0.05	3.16 ± 0.05
0.01	5.10 ± 0.19	2.36 ± 0.03	3.22 ± 0.04
0.05	6.21 ± 0.24	2.53 ± 0.01	3.48 ± 0.01

Table F. T_1 relaxation time of mixed methionine-6PG solutions with increasing concentrations of Fe(II).

Fe ratio	4.19 ppm	4.09 ppm	3.96 ppm	3.85 ppm	2.63 ppm	2.16 ppm
0	2.33 ± 0.20	1.32 ± 0.10	0.88 ± 0.07	2.61 ± 0.06	2.33 ± 0.02	3.18 ± 0.01
0.005	1.18 ± 0.03	0.92 ± 0.02	0.69 ± 0.02	1.58 ± 0.03	1.94 ± 0.02	2.64 ± 0.02
0.01	1.02 ± 0.03	0.84 ± 0.02	0.65 ± 0.02	1.42 ± 0.02	1.84 ± 0.02	2.50 ± 0.03
0.05	0.89 ± 0.06	0.78 ± 0.03	0.66 ± 0.02	1.19 ± 0.03	1.63 ± 0.03	2.24 ± 0.05

Table G. T_1 relaxation time of G6P solutions with increasing concentrations of Fe(II).

Fe ratio	5.22 ppm	3.99 ppm	3.92 ppm	3.87 ppm	3.70 ppm	3.56 ppm	3.50 ppm	3.27 ppm
0	4.02 ± 0.21	0.98 ± 0.03	0.93 ± 0.01	2.02 ± 0.06	4.06± 0.19	1.80± 0.02	2.70 ± 0.03	4.11 ± 0.08
0.005	3.60 ± 0.50	0.73 ± 0.02	0.73 ± 0.02	1.47 ± 0.08	3.02 ± 0.22	1.53 ± 0.06	2.10 ± 0.06	3.27 ± 0.14
0.01	3.85 ± 0.14	0.80 ± 0.04	0.78 ± 0.03	1.61 ± 0.06	3.31 ± 0.13	1.62 ± 0.04	2.20 ± 0.09	3.50 ± 0.14
0.05	4.19 ± 0.39	1.03 ± 0.01	0.92 ± 0.02	1.64 ± 0.10	3.50 ± 0.27	1.82 ± 0.03	2.42 ± 0.08	3.86 ± 0.16

Table H. T_1 relaxation time of mixed G6P-cysteine solutions with increasing concentrations of Fe(II).

Fe ratio	5.22 ppm	3.99 ppm	3.92 ppm	3.88 ppm	3.85 ppm	3.70 ppm	3.56 ppm	3.50 ppm	3.27 ppm	3.03 ppm
0	4.08 ± 0.70	1.25 ± 0.09	0.99 ± 0.02	8.41 ± 0.25	2.56 ± 0.24	4.80± 0.60	1.89± 0.09	2.81 ± 0.06	4.41 ± 0.39	3.05 ± 0.13
0.005	4.75 ± 0.40	1.06 ± 0.07	0.87 ± 0.03	3.44 ± 0.01	2.12 ± 0.13	4.27 ± 0.35	1.83 ± 0.09	2.45 ± 0.07	3.84 ± 0.19	2.50 ± 0.08
0.01	3.34 ± 0.44	1.10 ± 0.22	0.84 ± 0.09	2.56 ± 0.16	2.02 ± 0.32	3.79 ± 0.50	1.68 ± 0.20	2.15 ± 0.09	3.31 ± 0.35	2.11 ± 0.12
0.05	3.05 ± 0.19	0.92 ± 0.02	0.75 ± 0.01	1.47 ± 0.03	1.56 ± 0.03	2.81 ± 0.04	1.56 ± 0.03	1.71 ± 0.05	2.59 ± 0.07	1.79 ± 0.05

References

1. G. L. Ellman, Tissue sulfhydryl groups. *Arch. Biochem. Biophys.* **82**, 70–77 (1959).
